# Influenza A Virus RNA Polymerase Is Enriched on the Chromatin of Innate Immune Response Genes

**DOI:** 10.3390/v18101063

**Published:** 2026-09-24

**Authors:** Jia Yi, Jessica Morel, Mickaël Costallat, Nathalie Lejal, Bernard Delmas, Christian Muchardt, Eric Batsché

**Affiliations:** 1 Computational, Quantitative, and Synthetic Biology (CQSB), Centre National de la Recherche Scientifique (CNRS), Sorbonne Université, 75005 Paris, France; yijia20161@gmail.com (J.Y.); michaelcostallat@gmail.com (M.C.); christian.muchardt@sorbonne-universite.fr (C.M.); 2 UMR 7238 CNRS, Institut de Biologie Paris-Seine (IBPS), Sorbonne Université, 75005 Paris, France; 3 Doctoral School Life Science Complexity (ED515), Sorbonne Université, 75005 Paris, France; 4 Unité de Virologie et Immunologie Moléculaires (VIM), UMR 0892, UVSQ, University Paris-Saclay, INRAE Centre de Recherche Île-de-France–Jouy-en-Josas–Antony, 78352 Jouy-en-Josas, France; jessica.morel@inrae.fr (J.M.); nathalie.lejal@inrae.fr (N.L.); bernard.delmas@inrae.fr (B.D.); 5 Doctoral School Structure and Dynamics of Living Systems (ED577), University Paris-Saclay, 78352 Jouy-en-Josas, France

**Keywords:** influenza A virus RNA polymerase, chromatin, promoter, enhancer, transcriptional termination, defense genes, H3K27ac, epigenetic, downstream-of-gene transcripts (DoGs)

## Abstract

The influenza A virus hijacks host cell machinery by “snatching” molecular caps from cellular RNAs to prime its own transcription. While this process is typically associated with the beginning of genes, this study reveals that the viral RNA polymerase exerts a far-reaching influence on host gene expression. Using genome-wide mapping and comparative pathway analyses, we show that viral polymerase (FluPol) recruitment is decoupled from general host transcriptional activity; instead, FluPol is markedly enriched at a distinct subset of promoters and enhancers associated with defense-related pathways. Beyond the initiation site, FluPol remains associated with host transcripts throughout elongation, extending into downstream-of-gene (DoG) regions. It further accumulates at gene ends, potentially contributing to widespread defects in transcription termination. By remaining linked to host transcripts across the entire gene body, FluPol could extend its opportunity for cap-snatching and impair the recycling of cellular transcription machinery. Together, these findings demonstrate how FluPol accumulation across key cellular defense loci spans from initiation through to termination, driving host transcriptional attenuation.

## 1. Introduction

Influenza A virus (IAV) is an RNA virus whose replication occurs in the nucleus of infected cells [1]. Viral transcription depends on the activity of host RNA polymerase II (RNAPII), a feature that is unusual among RNA viruses [2]. This dependence arises from the activity of the viral RNA polymerase complex (FluPol), composed of the PA, PB1 and PB2 subunits. FluPol initiates viral transcription using short RNA primers generated by endonucleolytic cleavage of the 5′ ends of capped host transcripts by the PA subunit [3,4,5]. This process, known as “cap snatching”, allows FluPol to produce viral translation-competent mRNAs by combining the first 10–15 nucleotides from a capped host RNA with a viral RNA sequence generated by transcribing the negative-sense genomic vRNA (−). Deep sequencing of the host-encoded portion of these chimeric mRNAs has indicated that about half of these sequences match the 5′ ends of mRNAs [6] or snRNAs, largely in proportion to their transcription level [7].

Several observations suggest that FluPol steals capped RNAs in the early phases of transcription elongation, close to the promoters, in the region where RNAPII is pausing before productive elongation [8]. Firstly, FluPol preferentially interacts with RNAPII phosphorylated at the serine-5 (S5p) within the repeated motifs of the C-terminal domain (CTD) [9,10,11,12,13]. Analysis by mNET-seq of viral RNAs co-immunoprecipitated with RNAPII has further shown a 1000-fold enrichment in S5p-RNAPII compared to the total pool of RNAPII, suggesting that the FluPol generating the vRNA segments requires the association with S5p-RNAPII [14]. This form is enriched at promoters becausethe S5 phosphorylation is associated to the the capping reaction, a required maturation step preceding the elongation of nascent RNAs [15,16,17,18]. Beyond the S5p-CTD tail, FluPol also engages the RNAPII stalk subunit RPB4 [19], a contact presumably occurring before RNAPII engages in processive elongation, while also interacting with a broader set of promoter-enriched factors including co-regulators, elongation factors, chromatin remodelers, and the nuclear RNA exosome [13,20,21,22,23,24,25,26,27]. This suggests that its association with the transcription machinery extends beyond S5p-CTD to firmly anchor the viral polymerase to active host promoters. Cap-snatching in the early phases of transcription also provides an attractive explanation for the coupling of vRNAs synthesis with the activity of RNAPII [10], ensuring that FluPol can find the capped-RNA primers. Existing observations suggest only minimal or no specificity in the promoter selection for cap-snatching sites. The higher frequency observed at highly transcribed genes is therefore attributed to the greater abundance of capped RNAs at these loci, including mRNAs, promoter-associated antisense capped small RNAs (PROMPTs), U1/2/4/5/7 snRNAs, and snoRNAs [6,7,26,28].

Some observations suggest, however, that the model of “non-specific, promoter-only targeting” may not fully capture the complexity of the interactions between the viral and cellular transcription machineries. First, the limited depth of RNA sequencing and the challenge of unambiguously mapping the very short snatched host primers (10–15 nucleotides) could introduce significant bias, potentially explaining, at least in part, the apparent tropism of FluPol for highly expressed genes. Next, while S5p-RNAPII is enriched at promoters, it is not absent from gene bodies. Likewise, capping enzymes are not only recruited at transcription start sites (TSSs) but are also present inside genes, at the transcriptional termination sites (TTSs) and in the 3′ downstream flanking regions [29]. Capping factors can therefore generate capped RNAs not only at promoters but also at any site of new transcription initiation, including short non-coding pervasive transcripts and enhancers [30,31,32,33]. Indeed, enhancers are far more abundant in the genome than canonical promoters. Although individual enhancer RNAs (eRNAs) are generally short-lived, they are systematically 5′-capped [34], thereby representing a vast, alternative reservoir of nascent capped transcripts for the FluPol. Thus, if the sole purpose of FluPol recruitment to chromatin is cap-snatching, then a broader distribution targeting not only promoters but also active enhancer loci and gene bodies would be expected.

Furthermore, the functional impact of cap-snatching on host transcription extends beyond the mere harvesting of capped primers. Cleavage of the nascent transcript and loss of the 5′ cap at promoter-proximally paused RNAPII are known triggers for early transcription attenuation. Recent works, notably by Adelman and colleagues, have demonstrated that uncapped or cleaved nascent pre-mRNAs at paused RNAPII provoke premature transcription termination mediated by the Integrator complex [35,36]. By stripping the 5′ cap, FluPol activity likely induces an extensive, premature, wave-like termination event in the pausing window—presumably via Integrator/INTS11 endonuclease activity—which dramatically restricts the flux of RNAPII entering productive elongation. This widespread attenuation near the TSS overloads early termination machineries and inherently leads to a marked depletion of the RNAPII signal across gene bodies.

Finally, the cap-snatching activity of FluPol is regarded as a key component of the viral strategy to evade host cell defense and drive host transcriptional shutoff, as the loss of the 5 cap destabilizes host mRNAs and severely impairs cellular protein synthesis [37,38]. Yet, in many strains, IAV also causes global termination defects through the inhibition of transcriptional termination complexes by its NS1 factor [14,39,40,41], a capacity that remains strain-dependent and evolutionarily adaptable [42]. This disruption causes RNAPII to undergo long run-through transcription events in intergenic regions, effectively trapping the polymerase on chromatin and preventing its re-initiation at promoters. Such extended transcription results in the production of downstream-of-gene (DoG) RNAs, a phenomenon also observed in cells under stress [43,44]. Like cap-snatching, this strategy also occurs on chromatin and involves transcription machinery. It is tempting to speculate that these seemingly distinct phenomena—Integrator-mediated early attenuation triggered by cap-snatching at the 5′ end and NS1-driven read-through at the 3′ end—represent two coordinated facets of a unified viral strategy to paralyze host transcription.

We therefore deemed it necessary to reinvestigate the crosstalk between the host and viral transcription machineries. To this end, we monitored the dynamics of chromatin-associated transcripts during IAV infection while simultaneously tracking the recruitment of FluPol to chromatin. For the latter, we performed a series of chromatin immunoprecipitation experiments followed by deep sequencing (ChIP-seq) using different antibodies targeting the FluPol subunits PA, PB1 and PB2. We also utilized a FluPol PA subunit labeled with a highly efficient V5 epitope to perform ChIP assays and analysis of chromatin-bound RNAs. These approaches unexpectedly revealed that FluPol remains associated with nascent transcripts (likely through its interaction with RNAPII) during transcriptional elongation and termination. Furthermore, we discovered that genes recruiting FluPol to their TSS, TTS, gene body, or enhancers were more frequently involved in cellular defense mechanisms than would be expected by chance. Thus, our findings collectively indicate that FluPol disproportionately accumulates across virus-activated genes, from their TSS to their downstream 3′ end region. We therefore propose that FluPol impacts host cell transcription not only during the initiation phase but also throughout elongation and termination, which thereby amplifies its suppressive effect on host cell defense mechanisms.

## 2. Materials and Methods

### 2.1. H1N1 Virus Construction and Preparation

This study utilized Influenza virus A/WSN/1933 (H1N1). Both wild-type and V5-tagged PA versions of the virus were generated using the 12-plasmid reverse genetics system, as previously described [45]. The V5 epitope (amino acid sequence: NH_2_-GKPIPNPLLGLDST) was fused in-frame at the 3′ end of the PA gene, connected through a three-glycine linker. To create the PA-V5 plasmid used for virus rescue, a silent *NcoI* restriction site was introduced at nucleotide positions 2116–2121, which enabled the incorporation of exogenous sequences into the 3′ domain of the PA gene. To preserve the PA viral incorporation signal, the PA-V5 coding sequence was followed by a stop codon and the final 116 nucleotides (3′ end) of the PA segment.

### 2.2. Cell Culture and H1N1 Infection

A549 cells (CCL-185) were obtained from ATCC (Manassas, VA, USA) and cultured in Dulbecco’s Modified Eagle’s Medium (DMEM; Gibco, Waltham, MA, USA) supplemented with 7% (*v*/*v*) fetal bovine serum (FBS; Thermo Scientific, Waltham, MA, USA) and 100 U/mL penicillin–streptomycin (Gibco). For infection experiments, A549 cells were infected with either the V5-tagged PA H1N1 virus (virusV5) or wild-type WSN H1N1 virus at a multiplicity of infection (MOI) of 5 for 6 h post-infection (h.p.i.). Cells were either unfixed or fixed using 0.5%, 1%, or 2% formaldehyde in PBS for 10 min at room temperature prior to harvesting. The cell pellets were subsequently frozen at −80 °C until extract preparation.

### 2.3. Antibodies

The following antibodies were used in this study: H3K27ac (C15410196) and H3K4me1 (C15410037) from Diagenode (Liège, Belgium); pS5 RNAPII for Western blot (Ab5131), pS5 RNAPII (CTD 4H8) and unphosphorylated RNAPII (8WG16) for ChIP and histone H3 (Ab1791) from Abcam (Cambridge, UK); PA (PA5-31315), PB1 (PA5-34914), and PB2 (PA5-32220) from Thermo Fisher Scientific (Waltham, MA, USA); and anti-V5 epitope (A190-120A) from Bethyl Laboratories (Montgomery, TX, USA).

### 2.4. Chromatin Immunoprecipitation (ChIP and RNA-ChIP) and RNA–Chromatin Extraction

ChIP assays were performed as previously described [46,47]. Cells were crosslinked for 10 min at room temperature in PBS containing 1% (*v*/*v*) formaldehyde, followed by a 5 min wash in PBS with 125 mM glycine before harvest. Resuspended cells (10–20 × 10^6^ in 1.5 mL µtube) were incubated on ice for 5 min in 1 mL of chilled buffer A (0.25% Triton, 10 mM Tris pH 8, 10 mM EDTA, 0.5 mM EGTA), followed by 30 min in 1 mL of buffer B (250 mM NaCl, 50 mM Tris pH 8, 1 mM EDTA, 0.5 mM EGTA), and then resuspended in 100–200 µL of buffer C (1% SDS, 10 mM Tris pH 8, 1 mM EDTA, 0.5 mM EGTA) at room temperature. All buffers were freshly supplemented with 0.5 U/µL RNasin (Promega, Madison, WI, USA), Complete protease inhibitor, and PhosSTOP phosphatase inhibitor (Roche, Basel, Switzerland). Cell suspensions in 0.6 mL µtubes were sonicated in a water bath at 4 °C for 12 min (15 s ON, 15 s OFF) using a Bioruptor apparatus (Diagenode, Liège, Belgium) set to high power, followed by clarification through 10 min centrifugation at 10,000 rpm at 4 °C. DNA shearing was verified after reversing crosslinking by agarose gel electrophoresis, yielding fragments of approximately 300–500 bp. The sheared chromatin was quantified by optical density at 260 nm and diluted 10-fold in IP buffer to final concentrations: 1% Triton, 0.1% sodium deoxycholate, 0.1% SDS, 150 mM NaCl, 10 mM Tris pH 8, 1 mM EDTA, 0.5 mM EGTA, 1 U/µL RNasin, and 1× protease and phosphatase inhibitors. For ChIP-PCR assays, 30 µg of chromatin and 2 µg of antibodies were incubated in a final volume of 500 µL at 4 °C for 16 h on a wheel. For ChIP-seq or RNA-ChIP-seq assays, 200 µg of chromatin was incubated with 10 µg of antibodies in a final volume of 2 mL. To recover the immunocomplexes, 20 µL of saturated magnetic beads coupled to protein G (Dynabeads, Thermo Fisher Scientific) per µg of antibody were used. After 2 h of incubation, the bound complexes were extensively washed on a wheel for 10 min at room temperature with the following wash buffers: WBI (1% Triton, 0.1% sodium deoxycholate, 150 mM NaCl, 10 mM Tris pH 8), WBII (1% Triton, 1% sodium deoxycholate, 150 mM KCl, 10 mM Tris pH 8), WBIII (0.5% Triton, 0.1% sodium deoxycholate, 250 mM NaCl, 10 mM Tris pH 8), WBIV (0.5% Igepal CA-630 (Sigma, St. Louis, MO, USA), 0.5% sodium deoxycholate, 250 mM LiCl, 10 mM Tris pH 8, 1 mM EDTA), WBV (0.1% Igepal, 150 mM NaCl, 20 mM Tris pH 8, 1 mM EDTA), and WBVI (0.001% Igepal, 10 mM Tris pH 8). Input chromatin (20 µg) and ChIP beads were boiled for 10 min in 100 µL H_2_O containing 10% (*w*/*v*) Chelex resin (Bio-Rad, Hercules, CA, USA), followed by digestion with 0.2 mg/mL Proteinase K at 55 °C for 30 min and a final incubation at 100 °C for 10 min. Subsequently, 0.5 µL of the prepared DNA was used for quantitative real-time PCR assays. For RNA–chromatin extraction, the supernatant from the sonication step was purified using the acidic phenol/chloroform method and ethanol-precipitated with 5 µg of GlycoBlue (Ambion, Thermo Fisher Scientific) as a carrier. After resuspension, the input samples and the eluates from RNA-ChIP were treated with 5 U of TURBO DNase (Ambion) for 1 h at 37 °C. Subsequently, the RNA was purified again using the acidic phenol/chloroform method and ethanol precipitation. The resulting RNA samples were then tested for DNA contamination by qPCR using 1 µL of the eluate and primers targeting genomic repetitive elements. If the qPCR cycle threshold (Ct) was below 35, an additional round of DNase digestion and purification was performed to ensure the complete removal of DNA contamination.

### 2.5. Library Preparation and High-Throughput Sequencing

We used 1 to 5 nanograms of immunoprecipitated DNA to build DNA libraries. Libraries were prepared following the manufacturer’s instructions forNEBNext^®^ Ultra™ II DNA Library Prep Kit for Illumina^®^ (NEB #E7645L) and indexes (NEB #7335S; #E7500S; #E7710S). No size selection was performed. The quality and size of the libraries were assessed using the 2100 Bioanalyzer (Agilent Technologies, Santa Clara, CA, USA). Input DNA and the resulting libraries were quantified using a Qubit fluorometer (Invitrogen, Thermo Fisher Scientific). Indexed libraries were pooled according to the Illumina calculator (https://support.illumina.com/help/pooling-calculator/pooling-calculator.htm, accessed on 15 January 2020) and sequenced on a Illumina platform provided by Novogene (HiSeq-SE50).

For chromatin-associated RNA-seq, independent biological triplicates were processed for both mock and infected conditions. Purified Input RNA was quantified using a NanoDrop spectrophotometer, whereas low-yield RNA-ChIP samples were quantified using the Qubit RNA HS Assay (Thermo Fisher Scientific). Purified chromatin-bound RNAs exhibited fragment sizes ranging predominantly between 100 bp and 500 bp. For V5-PA RNA-ChIP in infected cells, individual biological replicates yielded ~125–200 ng of RNA, all of which was used for library preparation. Because RNA yield from IgG control ChIP was near the detection limit (~30 ng per replicate), equal amounts of RNA from three independent biological replicates were pooled and concentrated to obtain ~100 ng of total input RNA. RNA-seq library preparation and sequencing were conducted by the GenomEast platform, part of the ‘France Genomique’ consortium (ANR-10-INBS-0009) at IGBMC, Illkirch, France. Libraries were generated using 100–300 ng of RNA with TruSeq Stranded Total RNA Library Prep Gold kit and TruSeq RNA Single Indexes kits A and B (Illumina, San Diego, CA, USA) according to manufacturer’s instructions. Briefly, ribosomal RNA was removed by Ribo-Zero beads. Chemically fragmented RNA was reverse-transcribed with random primers, followed by second-strand synthesis using DNA Polymerase I and RNase. The resulting cDNA underwent end repair, adapter ligation, and amplification by 12 cycles of PCR. Residual primers were removed by AMPure XP beads. Libraries were sequenced as paired-end 100-base reads on an Illumina HiSeq 4000. Image analysis and base calling were performed using RTA 2.7.7 and bcl2fastq 2.17.1.14.

### 2.6. Data Availability

ChIP-seq, RNA-ChIP-seq, and RNA-seq data have been deposited in the NCBI Gene Expression Omnibus database under GEO accession number GSE218084.

### 2.7. Protein Extraction for Immunoprecipitation

Frozen cell pellets corresponding to 1 × 10^7^ cells were thawed on ice and resuspended in 500 µL of buffer A supplemented with protease inhibitors (Roche). A 150 µL aliquot of the suspension was mixed with 180 µL of buffer B and subjected to sonication for 5 min at 4 °C using a BioRuptor(Diagenode) (high power, 15 s. ON/15 s. OFF). The resulting clarified supernatants were quantified by optical density (260 nm). For immunoprecipitation, 20 µg of protein extract was incubated with 1 µg of the indicated antibodies for 16 h at 4 °C on a rotating wheel, followed by a 2 h incubation with 30 µL of anti-rabbit Dynabeads. Beads were washed five times in wash buffer WBV, resuspended in Laemmli buffer containing 100 mM DTT, boiled for 20 min and the resulting supernatant was analyzed by Western blot.

### 2.8. Western Blot

Denaturated proteins were resolved by SDS–PAGE using 4–12% Criterion XT Bis-Tris Protein Gel (Bio-Rad) and transferred onto a nitrocellulose membrane (Bio-Rad). The membranes were blocked in PBS containing 0.1% Tween-20 and 5% (*w*/*v*) non-fat milk, then incubated with the primary antibody diluted 1:1000, as indicated in the figures. After three 10 min washes in PBS–Tween (0.1%), the membranes were incubated for 1 h at room temperature with either anti-mouse StarBright Blue700 antibodies (1:3000 dilution, Bio-Rad) or TrueBlot HRP anti-rabbit antibodies (1:2000 dilution). Following four additional 10 min washes in PBS–Tween (0.1%), the membranes were developed using chemiluminescence (for HRP detection) and quantified with the ChemiDoc MP imaging system (Bio-Rad).

### 2.9. Real-Time Quantitative PCR (qPCR)

Quantitative real-time PCR (qPCR) was conducted using 1 µL of ChIP eluate in a total reaction volume of 10 µL. Reactions were prepared with Brilliant III Ultra-Fast SYBR Green Mix (Agilent) and performed on a Stratagene MX3005p system (Agilent). Data analysis was carried out using MxPro software (v4.10 Build 389, Schema85), with the sequences of primers used listed in Table S7. Statistical analysis and graphical representations were generated using Microsoft Excel.

### 2.10. Bioinformatic Analysis

The human reference genome used for this study was Homo sapiens hg19 primary assembly obtained from Ensembl. Reads alignment was performed, and resulting SAM files were converted to BAM format, sorted by coordinate, and indexed using samtools (v1.7) [48]. For ChIP-seq data, including those generated in this study, data from MDM cells (GSE103477) [41] and the A549 H3K4me3 ENCODE track (GSE91218) reads were aligned to hg19 using bowtie2 (v2.3.4) [49] with the parameters: [-N 0 -k 1 –very-sensitive-local]. Only reads with a MAPQ score of 30 or higher, corresponding to uniquely mapped reads, were retained for subsequent analysis. Indexed BAM files were processed with MACS2 (v.2.1.1) [50] to identify peaks, using the parameter: [-p 0.05]. For RNA–chromatin and RNA-ChIP-seq datasets, reads were mapped to hg19 using STAR (v2.6.0b) [51] with the parameters: [--outFilterMismatchNmax 1 --outSAMmultNmax 1 --outMultimapperOrder Random --outFilterMultimapNmax 30]. Detailed sequencing read counts and alignment statistics for both human (hg19) and viral (H1N1) genomes are provided in Table S8. Data visualization was performed with the Integrative Genomics Viewer [52] utilizing the CPM-normalized BigWig files generated from BAM files with the Deeptools suite (parameter: --normalizeUsing CPM) [53].

Gene expression from chromatin–RNA (this study) was quantifiedusing featureCounts (v1.28.1) [54] from the Rsubreads package with the parameter: [-s 2] to account for strand specificity. The resulting read counts were analyzed for differential expression (DGE) using the DESeq2 package [55]. DESeq2 performed normalization, dispersion estimation and the statistical testing using its default parameters. Raw *p*-values were adjusted for multiple testing according to the Benjamini and Hochberg (BH) procedure, and genes with an adjusted *p*-value below 0.05 and a fold change exceeding 50% were considered differentially expressed. Additionally, raw counts generated by featureCount were used to produce normalized gene levels. This was achieved by converting mRNA counts to rpkm (Reads Per Kilobase of transcript per Million mapped reads) using the library total count of each sample and gene lengths, defined as the combined exons length for Ensembl gene. The average level of genes with half-life greater than 15 h as defined in [56] and expressed higher than 80% of all expressed genes (417 genes with chromatin–RNA levels exceeding 300 rpkm and 281 genes with more than 17 rpkm in total RNA extract from A549 cells mock or infected by H1N1 at 12 hpi (GSE103604) [40]) was used to calculate a corrective index applied at the gene level in infected cells. Moreover, to evaluate the correlation of differential gene expression between our chrRNA-seq and independent external total RNA-seq datasets from A549 cells infected at 8 hpi and 15 hpi, respectively, we analyzed GSE82232 [37] and GSE120183 [38] using DESeq2 package. To account for genes induced by infection, such as defense genes, and to avoid artificially reducing their level by averaging between mock and infected cells, the maximum mRNA level observed between these conditions was used. This approach was applied to generate the MA plot and to stratify gene expression quartiles as described in Section 3. Differential expression analyses were conducted on a curated set of 32,344 Ensembl-annotated genes (comparable in size to the DESeq2 analysis). Because Influenza A virus-induced inhibition of transcription termination causes pervasive RNAPII read-through across gene bodies, establishing an absolute minimum threshold to separate low-level transcription from background noise is challenging. Therefore, an inclusive approach was maintained by retaining all genes with at least one read and excluding only the 3000 lowest-expressed genes. *p*-values were calculated using a Student’s *t*-test (*n* = 3) and adjusted for multiple testing by applying the Benjamini and Hochberg procedure to determine the False Discovery Rate (FDR). Genes with an FDR below 0.05 and a fold change exceeding 50% were classified as differentially expressed.

Signal evaluation of ChIP-seq, RNA-ChIPseq and RNA-seq on defined windows was conducted as follows. Multiples regions based on Ensembl annotations (hg19, version 87) were generated using the bedtools suite (v2.27.1) [57]: 500 bp around the TSS, 500 bp around the center of the intron closest to the gene’s center, 500 bp around the TTS, from TTS to 1 kb downstream of the gene, from 10 to 11 kb downstream of TTS and from 40 kb to 41 kb downstream of TTS. For TSS regions, windows were retained only if they were at least 10 kb away from both extremities of other genes. For downstream regions of genes, only regions not overlapping and at least 10 kb away from any other gene from the TTS to the end of the counting region were retained. The counting tracks were converted to the SAF format and used as annotation files in featureCounts (v1.6.1), with parameters set to: [-s 2] for RNA-ChIP-seq or [-s 0] for ChIP-seq. Regions were classified as differentially enriched between infected and mock cells based on criteria outlined in each figure. Metagene profiles of RNA-ChIP-seq were generated using CPM-normalized BigWig files. To minimize technical noise, repetitive elements and highly expressed genes generating abnormal background artifacts were excluded prior to plot generation. ChIP-seq was conducted on the 3 FluPol subunits in duplicate. For regions of non-recruitment, a pseudo-count was introduced prior to computing the ratio to avoid undefined values. Significant changes were evaluated by considering the confidence interval range as specified in the figure legends. To increase statistical power for discerning the loci with significant FluPol recruitment ChIP-seq signals for PA and PB1 (*n* = 4), we integrated the ChIP-seq datasets for PA and PB1. A genomic region was defined as FluPol-enriched if it met the significance threshold in the PA dataset or the PB1 dataset or when considering the pooled reads from both subunits. This computational combination was used to provide a high-confidence occupancy map, with the acknowledgment that subunit accessibility to antibodies can vary depending on the local chromatin context or complex orientation.

Profiles of ChIP-seq recruitment were generated with computeMatrix and plotProfile functions, applied to BigWig files of fold change between infected and uninfected cells (with a pseudo-count). Ratios of unexpressed genes were used as a reference and set to 0. To assess H3K27ac signal at potential enhancer locations in ChIP-seq data from infected cells, H3K27ac peaks within 10 kb windows around promoters regions defined by A549 H3K4me3 peaks from ENCODE (GSE91218) were first excluded using bedtools intersect (v2.27.1). The remaining H3K27ac peaks and the successive 1 kb regions around them (up to 4 kb maximum) were used as counting tracks for featureCounts (v1.6.1) with default parameters for paired-end data. The levels of H3K27ac were normalized relative to the ChIP-seq signal obtained for H3. Statistical analysis and graphs were generated using Microsoft Excel.

Pathway analysis with gene names were performed with Enrichr [58]. Reported *p*-values were adjusted using the Benjamini–Hochberg method. For *p*-value adjustment, background genes were selected from the 32,344 genes expressed in A549 cells, with a subselection based on the specific comparisons performed (list of genes available in corresponding Supplementary Tables). Complete gene lists and selection parameters are provided in the corresponding Supplementary Tables. To assess pathway analysis of genes near enhancers, the proximal five genes within the regions extending 500 kbp downstream and 500 kbp upstream from H3K27ac peaks were selected, as described in the main text. Genes without any exon-coverage reads were excluded from the analysis. Gene lists were then submitted to the Enrichr website (https://maayanlab.cloud/Enrichr/, accessed on 15 December 2025) for pathway enrichment analysis using either KEGG pathway (2021), Molecular Signature DB (2020) or Gene Ontology (GO) Biological Process terms (2025), as detailed in the respective figure and table legends.

## 3. Results

### 3.1. High-Throughput Sequencing of Chromatin-Associated RNAs in IAV-Infected Cells Revealed Extensive Transcriptional Perturbations

To investigate the impact of influenza virus infection on host transcription without the confounding effects of RNA maturation, we sequenced chromatin-bound RNA in human lung epithelial A549 cells infected with an H1N1 influenza virus (A/WSN/33) at a high MOI (MOI = 5). At 6 h post-infection (hpi), we observed that up to 40% of genes within the upper three quartiles of the expression distribution were differentially regulated, as evaluated by the DESeq2 package. (Figure 1A and Figure A1A). Notably, the number of dysregulated genes was even higher than previously reported for total RNA extracts at this early stage of infection [14,40,41,59,60,61].

As previously reported, the strong perturbation of the cellular transcriptome induced by IAV infection led to a substantial redistribution of RNA-seq reads both among genes, between genes and in intergenic regions [14,40]. Such heterogeneous transcript distributions are known to impact the performance of statistical normalization methods [62,63,64]. To address this, we employed an alternative normalization method based on a set of reference genes. We reasoned that transcripts with a long half-life would remain largely unchanged after only 6 h of infection. To confirm this, we verified that the coupling between RNA stability and expression levels, as previously observed [56], is conserved in A549 cells. In both chromatin-associated RNAs and total RNA extracts [40], transcript abundance was tightly correlated with RNA half-life categories (R^2^ = 0.94 to 0.98 for total RNA and R^2^ = 0.92 to 0.96 for chromatin RNA in the long-lived transcript quartile; Figure A1B). Short-lived transcripts were enriched among lowly expressed genes, whereas long-lived transcripts consistently predominated in the highest expression quartiles. Thus, considering the 417 top 20% most expressed transcripts (>300 rpkm) with long half-lives (>15 h) [56], we found that approximately 40% of the top 75% most highly expressed genes were differentially regulated (Figure 1B and Figure A1C). However, there was a notable discrepancy compared to the genes identified using the DESeq2 pipeline, with only 77–80% overlap between the two analyses (Figure 1C and Table S1). The two normalization approaches yielded an almost identical rank-order profile across the entire transcriptome (Spearman’s rho = 0.9817, Figure A1F, left panel). This demonstrates that our reference gene strategy acts strictly as a global baseline recalibration, correcting the zero-centered threshold for differential expression calls during a global shutoff without altering the relative ordering or relative magnitude of gene expression changes.

To further evaluate whether our reference gene normalization introduces bias, we performed extensive cross-study comparisons with three independent IAV infection datasets: Bercovitch-Kinori et al. 2016 (8 hpi, total RNA) [37], Zhao et al. 2018 (12 hpi, total RNA) [40], and Gaucherand et al. 2019 (15 hpi, total RNA) [38] (Figure A1D). We observed that the 417 reference genes were predominantly represented in the upper expression quartiles of external datasets (>87%, Figure A1E), while fewer than 13% were differentially expressed. The only exception was the comparison with the Bercovitch–Kinori dataset, likely reflecting the use of only two replicates rather than three in other studies (Figure A1E). We calculated correlation coefficients (Spearman’s rho and Pearson’s determination coefficient r^2^) between our 6 hpi chrRNA-seq log2 fold changes and those from total RNA-seq datasets. Strong correlations for differentially expressed genes (rho = 0.65–0.83, r^2^ = 0.52–0.70, Figure A1F) demonstrate that chrRNA-seq fold changes at 6 hpi show a strong positive correlation with downstream total RNA dynamics at 8–15 hpi. Notably, an equivalent correlation (rho = 0.63) was reported when comparing total RNA data between studies [37,38]. Moreover, we identified significant overlaps of upregulated and downregulated genes with all three independent studies (Figure A1G). These overlaps were highly significant (hypergeometric exact test, Fisher’s *p* < 10^−13^) for all studies, with upregulated genes predominantly mapping to primary antiviral pathways, interferon signaling pathways, and innate immune activation (Figure A1H). This shows that transcriptional induction at the chromatin level at 6 hpi reflects the cytoplasmic accumulation of antiviral mRNAs at 8–15 hpi (Figure A1G). Genes altered exclusively in our chrRNA-seq (6 hpi) may reflect immediate, transient transcriptional responses occurring directly at the chromatin level before post-transcriptional processing, cytoplasmic transport, or mRNA turnover occur.

A gene ontology analysis of genes upregulated by at least four-fold (FDR < 0.05) upon infection in our chrRNAseq confirmed the expected enrichment in defense response pathways, as well as an enrichment of downregulated genes associated with transcriptional regulation (Figure A1I and Table S2).

Earlier studies have shown that IAV-dependent transcriptional termination defects result in the production of “Downstream-of-Gene” transcripts (DoGs) at many active genes [14,40,41]. Sequencing chromatin-bound RNAs proved highly effective in visualizing DoGs, with RNAPII terminal read-through observed at nearly all genes that rely on the canonical polyadenylation complex, including several genes previously reported as unaffected (*LY6E*, *APOL1*, *DEFB1* and *IFI6*) [40] (Figure A1J). Consistent with a role of the polyadenylation complex, and in agreement with earlier observations [14,65], replication-dependent histone genes did not produce DoGs (Figure A1K). This widespread termination defect likely contributes to the bias we addressed earlier, as we observed a general decrease in apparent exon coverage accompanied by an increase in read abundance beyond polyadenylation sites (Figure A1L and Figure 1D).

To further investigate the mechanisms disrupting transcription, we performed RNAPII ChIP on infected cells. This revealed that the decreased exon coverage in the RNA-seq data corresponded to reduced RNAPII accumulation at the promoters of highly expressed genes that were either unaffected (e.g., *GAPDH*, *RPLP0*) or induced (e.g., *IL8*) after 6 h of infection, as well as within the transcribed regions of moderately expressed genes (e.g., *CD44*, *CDH1*) (Figure 1E). These results align with the study by Chan et al. 2006 [20]. Conversely, we observed increased RNAPII accumulation at loci corresponding to genes not expressed in A549 cells, even after IAV infection, such as *H2BFWT*. This may result from read-through transcription originating from neighboring genes (Figure 1F). Re-analysis of publicly available ChIP-seq data from primary human monocyte-derived macrophages (MDMs) infected with IAV and harvested at 6 hpi [41] revealed a similar pattern: a decrease in RNAPII recruitment at expressed genes, accompanied by increased accumulation in intergenic regions and at genes not expressed in these cells (Figure A1M).

### 3.2. Quantification of Chromatin-Bound RNAs Confirms That the IAV Infection Induced Defects of Transcriptional Termination

We next reexamined DoGs using the improved detection enabled by the sequencing of the chromatin-associated RNAs. Genome browser visualization revealed that many genes produced DoGs even in the absence of IAV infection, underscoring the intrinsic generation of DoGs during the transcriptional termination process (see examples in Figure 1D and Figure A1K,M). Because the production of DoGs has been suggested to contribute to the global reduction in host cell transcription, we investigated the relationship between gene expression and DoG production. To evaluate changes in DoG production in infected cells, we quantified the density of oriented reads in 10 kb windows across a 40 kb region downstream of TTSs (Figure 2A). This quantification showed a robust overall upregulation of DoGs even at sites distal from the TTSs within the 40 kb region under scrutiny (Figure 2B, also illustrated in Figure A6 and Figure A7B). In total, half of the assessed downstream regions showed at least a 1.5-fold increase in transcription, while downregulation was observed in fewer than 1.5% of the regions (Figure 2C). Upregulated DoGs originated from upregulated genes in approximately 38% of the cases, which suggests that the production of DoGs does not per se prevent gene activation, at least during the early stages of infection. Yet, half of the upregulated DoGs were located downstream of genes unaffected by IAV infection (represented by the grey proportion of ‘up’ in Figure 2D), which strongly supports the previously suggested impact on termination. Finally, we surprisingly noted that approximately 10% of the upregulated DoGs, as well as 60% of the non-significantly modified DoGs, were associated with silent genes. Through visual verification, we were able explain this phenomenon by observing that these particular cases involved genes that were completely traversed by DoGs originating from neighboring genes.

In addition to these termination defects, we observed a distinct transcriptional perturbation characterized by a loss of the promoter-proximal pausing peak (consistent with RNAPII release or degradation) and a discrete accumulation of chromatin-associated RNA within the first intron (TSS to 10 kb), indicative of early transcriptional attenuation (Figure A2). This profile indicates that IAV infection also induces promoter-proximal or early-intronic transcript attenuation upstream of the downstream gene body processivity loss.

### 3.3. Genome-Wide Analysis Revealed FluPol Enrichment at Transcriptionally Active Chromatin to Suppress the Host Antiviral Response

We next explored the genome-wide recruitment of FluPol by chromatin immunoprecipitation sequencing (ChIP-seq). To ensure the specificity of our antibodies (anti-PA, -PB1, and -PB2), we first confirmed their ability to efficiently pull down the viral polymerase complex. All three antibodies co-immunoprecipitated pS5-RNAPII from infected cell extracts (Figure A3), which validates their efficacy in IP conditions and reflects the known interaction between FluPol and pS5-RNAPII [9,10,11].

To avoid interference from neighboring genes, we focused our analysis of the ChIP-seq data on genes located at least 5 kb away from other genes. Although overall DNA enrichment levels were relatively low—consistent with an indirect association with DNA mediated by RNAPII—the signals were highly specific to the infected condition. Profiles covering entire gene bodies, including introns (metagenes), of FluPol subunits indicated clear enrichment at the level of promoters (Figure A4A). Because our signals were normalized against non-infected (mock) cells with the same chromatin landscape, the observed enrichment could not be attributed to sonication bias or to the inherent accessibility of active genes. The examination of ChIP signals within 500 bp windows centered on promoter TSSs showed a significantly increase upon viral infection, which was more pronounced at expressed genes (defined by RNA-seq read counts) than at unexpressed genes (Figure 3A). Consistently, the signal was also higher at promoters marked by H3K27ac than at the TSS of unexpressed genes (compare the middle and the right panels in Figure 3A). Quantification confirmed that infection-induced enrichment at TSSs was significantly greater for all FluPol subunits at expressed genes than at unexpressed genes (above the red line, Figure 3B). We further noted that the combination of PA and PB1 signals improved the detection of expressed genes compared with either signal alone, which makes this combination a more effective proxy for FluPol recruitment (see methods, right bars in Figure 3B). By contrast, the anti-PB2 antibody performed relatively poorly in distinguishing infection-induced signals at expressed genes from those at unexpressed genes (Figure 3A,B and Figure A3). Finally, analysis of the intronic regions revealed no difference in the recruitment of FluPol subunits between expressed and silent genes (Figure A4B), indicating that FluPol enrichment was not detectable at random positions within gene bodies.

To further explore the relationship between FluPol recruitment and transcriptional activity, we analyzed the distribution of FluPol-enriched TSSs across quartiles of chromatin-associated RNA levels and of H3K27ac levels. While the proportion of TSSs with significant FluPol enrichment increased modestly across quartiles of chromatin-associated RNA levels, rising from 10% in the lowest to 18% in the highest quartile (Figure A4C), a stronger bias toward TSSs with the highest H3K27ac content was observed, where 25% of the top quartile showed significant FluPol occupancy, compared to only 6% in the lowest quartile (Figure A4D).

To examine whether FluPol recruitment could be explained by host gene expression levels, we assessed the correlation between FluPol significant ChIP enrichment (in infected cells) and transcriptional output in uninfected cells. Interestingly, we observed a very weak correlation (rho = 0.020) (Figure A4E), which indicates that FluPol recruitment is largely independent of baseline host gene expression. Furthermore, the correlation with transcriptional output in infected cells was even weaker and not significant (rho = 0.016). By contrast, we observed a moderate positive correlation between significant FluPol ChIP enrichment and H3K27ac levels at promoters in uninfected cells (rho = 0.394) and in infected cells (rho = 0.40) (Figure A4F). Together, these observations were consistent with the recruitment of FluPol subunits to sites of transcriptional initiation, with a particular preference for highly active promoters.

To determine whether FluPol recruitment is influenced by factors beyond transcriptional activity, we performed a pathway enrichment analysis on expressed genes where PA and PB1 were recruited to the TSS. This revealed a distinct functional signature characterized by a high frequency of infection-related pathways, such as ‘shigellosis’ and various viral infection pathways, in the top 10 most significant results in particular (Figure 3C). Notably, these pathways were largely absent from the top enrichment results of highly expressed genes (Figure A4G). Furthermore, they were substantially less enriched among genes showing either high baseline H3K27ac levels or acute H3K27ac increase upon infection (Figure A4H,I). This overrepresentation of infection-related loci was evident even though the pathways identified in the FluPol-enriched gene list had higher *p*-values than the broader metabolic pathways dominant in the other gene lists. The consistent enrichment of infection-related categories nevertheless suggests that FluPol recruitment is disproportionately enriched at genes involved in host–pathogen interactions.

To investigate the potential impact of FluPol recruitment on host gene expression, we examined the relationship between FluPol enrichment at gene TSSs and the differential expression of those genes during IAV infection. Surprisingly, upregulated genes showed the lowest enrichment for FluPol, which was instead significantly more prevalent at the TSSs of downregulated or unaffected genes (Figure 3D and Figure A4J). This observation supports the hypothesis that FluPol-mediated cap-snatching actively suppresses gene expression; specifically, while upregulated genes escape FluPol recruitment, downregulated genes exhibit high occupancy. Hence, the set of genes that did not show significant RNA changes might include those typically upregulated during the activation of cellular defense mechanisms but effectively suppressed by FluPol. Accordingly, FluPol recruitment was higher at TSSs with increased H3K27ac levels compared to those with stable or decreased acetylation (Figure 3E). Supporting these findings, we observed that FluPol-enriched genes were predominantly associated with pathogen infection pathways specifically when H3K27ac levels were increased or RNA levels were decreased (Figure 3F). By contrast, no significant pathway enrichment was found for genes characterized by decreased H3K27ac or increased mRNA levels (Figure A4K).

While FluPol recruitment requires host transcriptional machinery, it does not passively accumulate across all highly transcribed loci (Figure A4E). Rather than aligning with baseline promoter acetylation (Figure A4H,I), FluPol enrichment tightly tracks dynamic H3K27ac induction upon infection (Figure 3E,F), showing marked enrichment at chromatin undergoing active transcriptional activation. These data indicate that FluPol is more frequently associated with a hierarchical network of host defense initiators—specifically, primary sensors and transducers within the NF-κB/RELA and TBK1/IFNAR1 pathways (Table S3; exemplified at specific loci in Figure A4L). This accumulation of FluPol at the critical transition from recognition to activation provides a mechanistic framework for viral ‘host shutoff’, which may rely on specific interactions with activation-dependent co-factors, inducible transcription factors, or specialized RNAPII complexes.

### 3.4. FluPol Is Recruited to Enhancers in Regions Close to Immune Response Genes

Like promoters, enhancers are sites of RNAPII transcription and produce capped RNAs (eRNAs). We therefore investigated whether these regulatory elements might also serve as sites of FluPol recruitment. To this end, we identified a series of isolated enhancers active in infected cells, based on the presence of the H3K27ac histone, a mark characteristic feature of enhancers [66] and the absence of H3K4me3, which is enriched at promoters (Figure 4A and bioinformatics section in method). FluPol recruitment at these enhancers was then assessed by quantifying the ChIP-seq signal for each subunit within 1 kb bins surrounding the H3K27ac peak (Figure 4A, blue boxes). This analysis revealed that, upon infection, all FluPol subunits showed significantly increased accumulation within the first 1 kb from the center of the enhancer, with the signal rapidly decreasing as the distance from the center increased (Figure 4B—in the shown graphics, left and right windows are combined). Similar to promoters, the anti-PB2 antibody appeared to be the least efficient at detecting enhancers. Additionally, we noted that the recruitment of FluPol subunits to enhancers could be positively correlated with the level of H3K27 acetylation (Figure 4C). This suggests that FluPol enrichment at enhancers is dependent on enhancer activity.

We then examined the functional associations of enhancers bound by FluPol using genomic region enrichment analysis. For 508 enhancers showing high PA/PB1 occupancy (≥50% above background), we identified 3663 neighboring expressed genes within a 1 Mbp region centered on these peaks. KEGG pathway analysis revealed that these genes are predominantly involved in innate defense and viral infection signaling (Figure 4D left panel, Table S4A). This enrichment was specific to FluPol-occupied enhancers and was absent in FluPol-negative active enhancers (Figure 4D right panel, Table S4B). Visual inspection of the genomic data revealed such occupancy observed at the *CXCL* and *OAS* loci (Figure 4E). Collectively, these findings suggest that FluPol accumulation at specific enhancers may modulate the immune response, potentially by limiting their transcriptional activity.

### 3.5. FluPol Is Associated with Intragenic Chromatin-Bound RNAs

As an alternative approach to study FluPol recruitment to the host genome, we next implemented an RNA-ChIP approach to explore the range of chromatin-bound RNA species associated with the viral polymerase. Due to the insufficient quality of available antibodies for this demanding technique, we generated a V5-tagged version of the PA subunit of FluPol. The resulting recombinant H1N1-derived IAV retained its ability to infect A549 cells. To validate the efficiency and strict specificity of this system under RNA-ChIP conditions (see Section 2), we performed pull-downs on cells infected with either WT or V5-PA viruses. Anti-V5 immunoprecipitation resulted in robust recovery of the tagged V5-PA subunit, without background precipitation of WT PA or RNAPII in WT-infected control cells (Figure 5A and Figure A5).

In the control ChIP-PCR experiments, the recombinant FluPol containing V5-PA was efficiently detected at the DNA of promoters previously identified as recruiting the native version of the viral polymerase, including genes either genuinely expressed at moderate-to-high levels (*RPLP0*, *GAPDH*, *CD44*) or induced by infection (*IL8*) (Figure 5B). Notably, the more efficient immunoprecipitation achieved with the anti-V5 antibody revealed that V5-PA was also present within the coding regions of genes (CD44 and CDH1) (Figure 5C) as well as within a read-through region generated by runaway RNAPII that had initiated at promoters of upstream genes (H2BFWT) (Figure 5B and Figure 1F).

**Figure 5:**
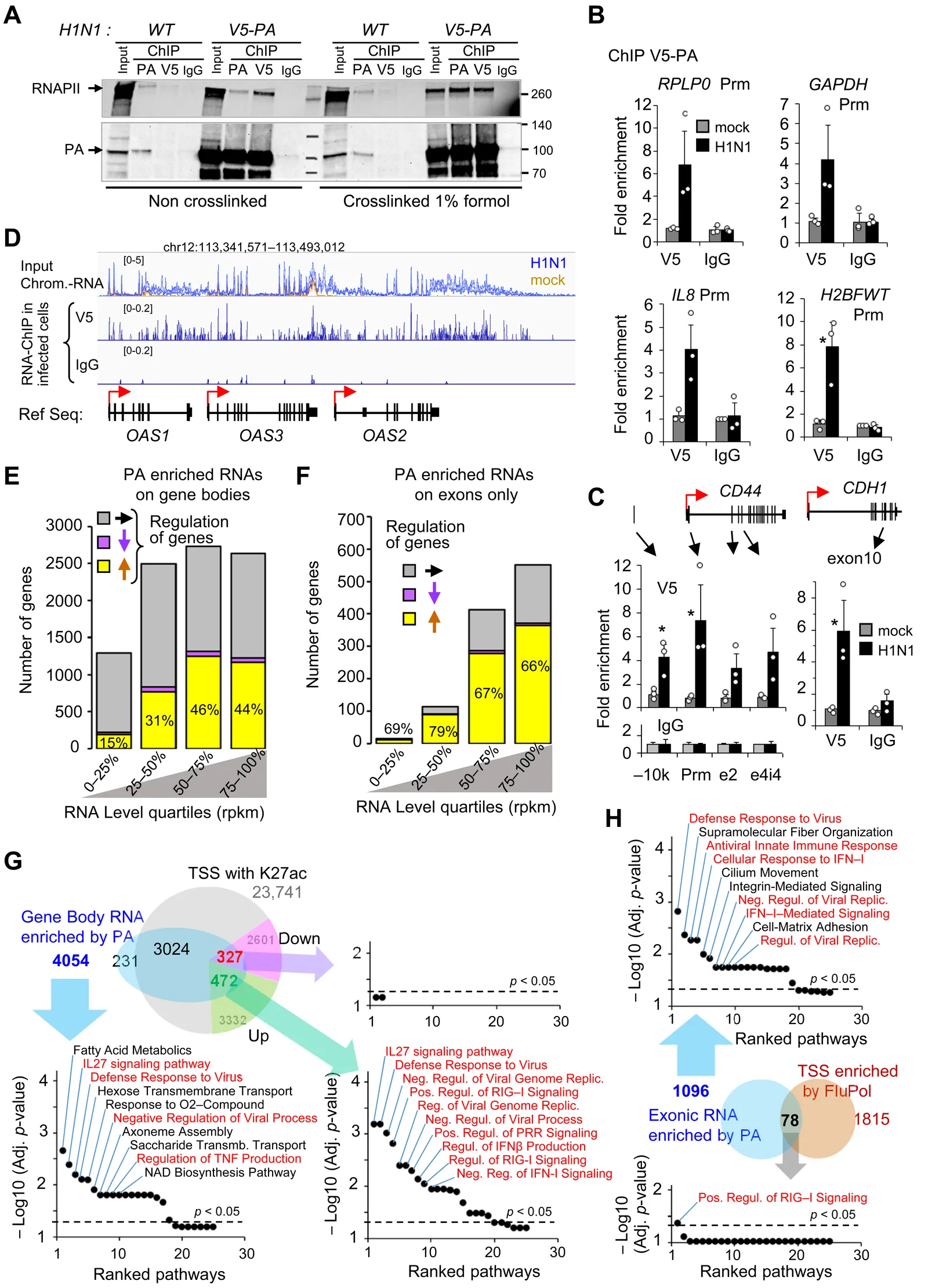
FluPol subunits on the chromatin are associated with nascent RNAs from the host cell and functionally enriched for antiviral genes. (**A**) A549 cells infected with either WT IAV or V5-PA-engineered IAV were crosslinked by 1% formaldehyde or left untreated. Chromatin fractions were subjected to immunoprecipitation with PA or V5 antibodies or non-immune IgG as a control. The eluates and 10% of the input extracts were resolved by Western blotting. The lower portion of the membranes was probed with PA antibodies, while the upper portion was probed with phospho-RNAPII antibodies. (**B**,**C**) ChIP assays were performed using anti-V5 and IgG antibodies from infected and uninfected cells. Immunoprecipitated DNA was quantified by qPCR with primers targeting the specified regions. Enrichment levels are expressed relative to the signal obtained from IgG ChIP in uninfected cells. Values represent the mean (±deviation) of three independent experiments. Statistical significance of differences was assessed by a two-tailed Student’s *t*-test, with *p* < 0.05 (*). (**D**) Example of PA-enriched RNAs bound to chromatin visualized using IGV. RNA-seq data from independent triplicates are overlaid in the same color for the (+) strand. The scale range for the tracks is indicated in brackets. The bottom track (RefSeq) displays the position of the genes and their orientations using red arrows. (**E**) RNAs from ChIP of V5-PA or non-immune IgG in V5-PA H1N1-infected cells were analyzed by deep sequencing. PA-enriched RNAs spanning gene bodies were evaluated and deemed significant if the mean change, adjusted by subtracting the triplicate CI (80%), was at least 1.5-fold greater than IgG controls. The number of enriched genes categorized as upregulated (yellow), downregulated (magenta) or unchanged (gray) was plotted as a function of gene expression levels (as determined in Figure 1B), with each quartile containing 8300 genes. (**F**) The same analysis as in (**E**) was performed for RNAs covering exons only. (**G**,**H**) Integration of PA-bound RNAs and H3K27ac promoter status. (**G**) Venn diagram comparing 4054 genes exhibiting at least a 20-fold enrichment of PA-bound gene body RNAs (vs. IgG) with genes showing differential H3K27ac levels at their transcription start sites (TSSs; top 90th percentile). Gene Ontology Biological Process analysis was performed for the total PA-enriched gene set (*n* = 4054), as well as for subsets with infection-induced H3K27ac elevation by more than 20% (*n* = 472) or reduction by at least 20% (*n* = 327) at the TSS. Pathways are ranked by adjusted *p*-value. The top 10 significantly enriched pathways are displayed, with defense-related pathways in red and others in black. (**H**) Venn diagram illustrating the overlap between the 1096 exonic RNAs enriched by PA (at least 1.5-fold vs. IgG, as defined in (**E**)) and the 1815 genes with FluPol-enriched TSS (as defined in Figure 3C). GO Biological Process analysis was conducted on the indicated gene sets. Notably, despite the relatively small number of overlapping genes, the RIG-I signaling pathway is significantly enriched among genes characterized by both PA-bound exonic RNAs and FluPol TSS occupancy. Full data are available in Table S5.

Implementing RNA ChIP-seq with V5-tagged virus and anti-V5 antibody revealed that chromatin-RNAs associated with V5-PA were extensively distributed across the coding region of a large set of expressed genes: a total of 9518 genes, corresponding to 32% of the examined genes (see example of the *OAS* genes, Figure 5D).

Quantification further revealed that immunoprecipitated chromatin-bound RNAs do not predominantly arise from the most highly expressed genes. Indeed, V5-PA targets genes in the lower expression quartiles as effectively as those in higher quartiles (Figure 5E). Metagene profiling further confirmed that the V5-PA RNA-ChIP signal is continuously distributed across the entire gene body and extends into the downstream region (Figure A6). We identified a subset of 1096 genes where V5-PA-bound RNAs were enriched for exonic sequences (Figure 5F). We refer to these as ‘spliced chromatin-RNAs,’ as they likely represent pre-mRNAs that have undergone full or partial co-transcriptional splicing. Interestingly, while the absolute number of V5-PA-bound spliced chromatin–RNAs increases with expression rank (Figure 5F), the fraction of infection-upregulated genes within this population remains strikingly constant across all quartiles (66–69%, compared to 15–46% for total chromatin-bound RNAs in Figure 5E). This uniform enrichment suggests that FluPol is disproportionately associated with upregulated genes that may undergo more rapid, co-transcriptional splicing.

Approximately 4050 genes exhibited more than a 20-fold enrichment relative to the IgG control for RNA attachment across gene bodies. The molecular signature of these enriched genes was highly significant for IFN pathways (Figure 5G, compare with Figure A1J), including targets such as OAS genes, DDX58/60, ISG15/20 and STAT3 genes (Table S5).

Furthermore, genes whose RNA was bound by PA and whose promoters were enriched for H3K27ac following infection (472 genes) significantly define pathways linked to cellular defense. By contrast, genes with PA-bound RNAs but H3K27ac-depleted promoters (327 genes) showed no significant pathway enrichment (Figure 5G, Table S5). A parallel analysis of the 1096 PA-bound ‘spliced chromatin RNAs’ revealed a similar clear enrichment for defense pathways (Figure 5H, top), with OAS, IFIT, ISG genes and DHX58/DDX60 among the targets in the most significant pathways. Notably, overlapping these exonic RNA targets with genes exhibiting FluPol-enriched TSSs (as defined in Figure 3C) revealed that the RIG-I signaling pathway remained significant (Figure 5H, bottom).

Together, these observations suggest a distinct association profile for FluPol, with an enrichment at genes upregulated by the infection rather than genes highly expressed before and after the infection. In particular, the association of FluPol with nascent and possibly partially mature mRNAs of the IFNα/γ pathway may contribute to the suppression of host cell defense mechanisms.

### 3.6. FluPol Is Associated with Chromatin-Bound RNAs Originating from Gene Termination Sites and Downstream Regions

We next used the V5-PA RNA ChIP-seq data to examine in more detail the association of FluPol with DoGs. The distribution of RNAs bound to the V5-PA subunit suggested enrichment at the 3′ end of genes (TTS) and at regions covered by DoGs (Figure 5D and Figure A7). This was verified quantitatively by counting reads in the regions extending 40 kb downstream of the TTSs of all genes, in 10 kb bins (Figure 6A). When reads were consolidated over the entirety of the 40 kb regions, the median density of V5-PA-associated RNAs was more than 200-fold (2^8^) higher than that observed in IgG negative control (Figure A7A). To further characterize this spatial distribution, we generated metaplots of read coverage across 40 kb of the 3′ extended region. V5-PA RNA-ChIP-seq signals showed a pronounced enrichment over IgG, closely matching the elevated input chrRNA-seq signal observed in infected compared to mock-infected cells (Figure A7B). Among genes containing upregulated DoGs (|FC-CI| > 1.5), the proportion of DoGs significantly associated with V5-PA (|FC-CI| > 1.2 versus IgG control) was four times higher than that observed in unmodified DoGs, with little or no representation of downregulated DoGs (0–2%) in the V5-PA-bound fraction (Figure 6B). This suggests that the FluPol may participate in the complex involved in inhibiting termination processes and could contribute directly to the mechanism causing the production of DoGs in infected cells.

To further confirm the presence of FluPol in the DoG regions, we analyzed the DNA ChIP-seq data obtained with the antibodies against FluPol subunits to quantify the enrichment of individual subunits at TTSs and downstream regions. To avoid interference from overlapping, neighboring or converging genes, we selected genes separated from each other by at least 10 kb. PA and PB1 were the most efficiently enriched in the downstream regions of expressed genes, with silent genes defining the background signal (Figure 6C). The enrichment of these FluPol subunits was, on average, strongest at regions proximal to the TTS (compare quantifications at 500 bp, 1 kb, 10 kb and 40 kb away from the TTS), which suggests that the coding regions and the proximal terminal downstream region were prominent sites for FluPol binding to nascent transcripts.

Analysis of the 1454 genes associated with PA-bound DoGs identified four of the top 10 significantly enriched pathways related to viral infections (coronavirus disease, hepatitis C, viral carcinogenesis, and hepatitis B). These pathways exhibited a molecular signature characteristic of IFN/STAT responses, including key genes such as *ISG20*, *NFKBIA*, *IRF7*, *IFI35*, *IFIH1*, *DDX58*, *ITGB7*, *TYK2*, and *STAT3*. By contrast, DoGs not enriched for PA showed no significant pathways and none directly linked to viral infections (Figure 6D, left panel and Table S6). When 1676 genes with FluPol enrichment at 1 kb post-TTS regions were examined, no pathways reached significance using adjusted *p*-values, which suggests broader chromatin association and statistical dilution. However, considering unadjusted *p*-values, the top 10 pathways included viral infection-related processes (JAK-STAT signaling, a cornerstone of IFN responses, legionellosis, and hepatitis B), with several genes related to the IFN pathway, such as *IFNGR2*, *IL6*, *IL1R1*, *IL3RA*, *TYK2*, and *STAT3/5A* (Figure 6D, right panel and Table S6). Notably, both gene sets shared “Non-alcoholic fatty liver disease” and “Pathways of neurodegeneration” among their top 10 pathways, which suggests the involvement of genes related to systemic inflammation or stress responses. More strikingly, the intersection of 118 genes common to both PA-bound DoGs and FluPol-enriched TTS regions revealed five infection-related pathways among the top 10 significant hits (adjusted *p*-values): EBV, CMV, HHV8, shigellosis, and HIV-1 infection (Figure 6D, central panel and Table S6). The convergence of these two different approaches suggests that FluPol’s TTS association is not random but rather is disproportionately concentrated at a core set of genes exhibiting marked termination disruption. These data indicate that physical binding does not always correlate with termination defects, but where it does, the affected genes are predominantly defense-related genes.

## 4. Discussion

Analysis of the chromatin compartment revealed the extensive impact of IAV infection on host transcription, which affects both gene activity (Figure 1C) and the genomic distribution of RNAPII (Figure 2). This inhibition of gene termination results in a general decrease in RNAPII at promoters and in gene bodies and an increase in intergenic regions (Figure 1D–F and Figure A1K–M). Our mapping of the three FluPol subunits suggests that the viral polymerase may contribute to these alterations. To our knowledge, this provides the first large-scale genomic footprint of FluPol, revealing its persistence far beyond the promoters and enhancers typically associated with cap-snatching, extending through gene bodies and transcriptional termination sites (TTSs) into downstream intergenic regions (Figure 3, Figure 4, Figure 5 and Figure 6).

While RNA abundance clearly influences cap-snatching efficiency, our data suggest that FluPol enrichment cannot be explained by overall transcription levels alone. Notably, genes exhibiting the most significant FluPol occupancy at their TSS are not those with the highest RNA levels but rather those whose expression is downregulated during infection (Figure 3D and Figure A4J). This inverse relationship argues against a simple model in which FluPol passively associates with the most abundant transcripts or the most active promoter. Instead, FluPol enrichment is most pronounced at promoters activated during infection, consistent with its strong association with defense-related pathways (Figure 3C,E,F). Together, these observations suggest that FluPol is disproportionately associated with infection-responsive genes.

The non-random nature of this association demonstrates that FluPol remains bound to host nascent transcripts and is markedly concentrated at DoG regions (Figure 5D–F, Figure 6A,B, Figure A6 and Figure A7). This same distinction between RNA abundance and chromatin occupancy is evident at the 3′ end, where FluPol association was not solely dictated by the most abundant DoGs (Figure 6D). Crucially, this enrichment is most pronounced at upregulated DoGs, which suggests a potential link between FluPol occupancy and the inhibition of terminal cleavage (Figure 6B).

We propose that maintaining proximity to the entire transcriptional unit could provide a dual advantage for the virus: it may optimize cap-snatching efficiency from nascent RNAs while simultaneously providing a spatial platform to interfere with host termination factors. In this model, FluPol complements the known inhibitory role of NS1, effectively suppressing the host response at the site of transcription initiation and termination. While this accumulation at defense-related loci could reflect an active viral enrichment mechanism or efficient co-localization at host-activated transcriptional hubs, this spatial positioning represents a key feature contributing to host shutoff. This expands the functional repertoire of FluPol beyond its established role in primary transcription initiation and provides a framework for future studies to dissect whether specific viral adaptors or host-driven chromatin opening dictate this association during infection.

### 4.1. Transcriptional Changes During Viral Infection Confound the Analysis of Differential Gene Expression

Before delineating the molecular mechanisms of virus–host interference, establishing a robust analytical framework is essential, as infection-induced global transcriptional shifts can severely confound differential gene expression (DEG) analyses. Indeed, it is well established that IAV infection induces terminal transcription defects, leading to the production of “downstream-of-gene” (DoG) transcripts (DoGs) [14,40]. While these DoGs are typically transient and rarely captured in standard RNA-seq, they become significantly elevated during IAV infection. This pervasive transcription poses a major challenge for accurate mRNA quantification; the extensive redistribution of RNA-seq reads across the genome violates the fundamental assumptions of standard differential expression algorithms, such as DESeq2 [62,63]. To address this, we employed a normalization method using reference genes with mRNA half-lives significantly longer than the duration of our infection observation window. This approach revealed that DESeq2-based assessments were uncertain for approximately 25% of genes (Figure 1C), with many of these discrepancies arising from the misassignment of gene-specific reads to DoGs originating from upstream loci (Figure 1D,F and Figure A1J,L).

To further validate our approach, we compared our chrRNA-seq data with external total RNA-seq datasets [37,38,40]. We observed moderate-to-strong correlations between our results and those of these independent studies, which likely arose from biological differences between nascent chromatin-associated transcription kinetics and total cytoplasmic RNA accumulation. Crucially, the bias observed between DESeq2 and our reference gene analysis may also stem from the subcellular fraction analyzed. While standard RNA-seq typically focuses on soluble polyadenylated RNAs from total extracts, our study specifically interrogates chromatin-bound RNAs. This fraction is enriched for nascent transcripts and transient read-through species (like DoGs) that may not yet be polyadenylated or accumulated in the cytoplasm. Thus, transcriptional changes at the chromatin level at 6 hpi reflect the cytoplasmic accumulation of antiviral mRNAs at 8–15 hpi (Figure A1G). Consequently, our method captures a more immediate snapshot of transcriptional disruption that standard “soluble” RNA-seq may overlook.

This redistribution of RNA is mirrored by a massive relocalization of RNAPII after viral infection, which can similarly bias ChIP-seq evaluations (Figure A1M and ref. [41]). We confirmed via ChIP-PCR that RNAPII accumulation within gene bodies is significantly decreased upon IAV infection (Figure 1E). Notably, our assessment of chromatin-bound RNAs yielded transcriptional profiles well-aligned with high-resolution techniques like NET-Seq [14]. Taken together, these findings emphasize that selecting an appropriate normalization strategy and considering the specific RNA fraction (chromatin vs. soluble) are critical when studying biological phenomena, such as viral infections that disrupt the global genomic distribution of both RNAs and RNAPII.

### 4.2. FluPol’s Chromatin Association and Cap-Snatching Temporal Extension

Having validated our normalization framework, we next investigated how host transcription is disrupted at the chromatin level. Our findings demonstrate that FluPol’s association with chromatin extends beyond transcription start sites (TSSs), challenging the conventional view that cap-snatching is exclusively confined to promoter regions. Although we observed varying efficiencies in ChIP using antibodies against native PA and PB1 subunits—and poor performance with anti-PB2—the significant enrichment of FluPol on transcriptionally active genes compared to silent loci confirms the reliability of our data (Figure 3 and Figure 6C). This observation is further supported by RNA-ChIP-seq experiments using an anti-V5 antibody to detect a V5-tagged PA variant (Figure 5A,B and Figure A5).

Previous studies have shown interactions between FluPol and the Ser5-phosphorylated (pS5) form of RNAPII [9,10,11,12], transcriptional coactivators [21,23,24,27], the chromatin reader CHD1 that recognizes the promoter-specific H3K4me3 marks [25], elongation factors [13,22] and the nuclear RNA exosome [26]. These interactions have led to the prevailing model that cap-snatching occurs primarily when nascent RNAs emerge from RNAPII paused at promoters [67]. However, our chromatin localization data suggest a more complex spatial context for this process. While not disputing FluPol occupancy at promoters, our results indicate that cap-snatching may not be restricted to polymerases awaiting promoter escape, which significantly expands the spatial context in which this reaction could take place.

In this extended context, the functional consequences of early 5′ cap-snatching on transcription elongation must also be considered. The cleavage and removal of the 5′ cap by FluPol leaves nascent transcripts vulnerable to early attenuation. Supporting this indirect mechanism, our chrRNA-seq profiling revealed a loss of the promoter-proximal pausing peak (consistent with RNAPII release or degradation) and a discrete accumulation of chromatin-associated RNA within the first intron (Figure A2). This phenotype may mirror the transcriptional attenuation defects reported upon Integrator complex disruption, particularly in the absence of INTS11, which has been shown to lead to increased DoG production [68]. Loss of the 5′ cap may thus trigger premature attenuation near the TSS—potentially by shifting or overloading Integrator-mediated termination machinery—thereby contributing to the widespread drop in RNAPII processivity across gene bodies alongside direct viral interference with 3′ processing factors.

A key limitation of our approach is that FluPol occupancy or association with chromatin-bound RNAs does not directly demonstrate active cap-snatching catalysis on these specific transcripts. Nevertheless, while direct evidence for downstream cap cleavage remains technically challenging to isolate, several lines of evidence support the hypothesis that this prolonged association provides a functional advantage. Interestingly, PA-associated RNAs show greater upregulation and more significant involvement in cellular responses when considering exonic sequences rather than entire gene bodies (Figure 5E–H). This suggests potential interactions between PA and chromatin-bound spliced RNAs, in addition to the 3′ end DoGs. During this process, co-transcriptional splicing allows partial RNA maturation before the complex reaches the transcription termination site (TTS). At the TTS, termination inhibition enables transcription to continue beyond the polyadenylation site, with FluPol remaining associated with both the elongating complex and the spliced mRNA (Figure 7). Beyond host transcript processing, the proximity to host machinery may facilitate the processing of viral genomic segments 1, 7, and 8, which require host factors for splicing (generating PB2-S1, M2, M42 and NS2 viral proteins), terminal cleavage, polyadenylation [69], and nuclear export [2,70].

In addition, this extended “window of opportunity” may explain why FluPol preferentially snatches caps from mRNAs rather than from enhancer RNAs (eRNAs) or promoter anti-sense RNAs (PROMPTs) [26]. Indeed, as we have shown FluPol localization on enhancers (Figure 4), it was reasonable to hypothesize that enhancers—being ten-fold more numerous than promoters [31]—could represent a significant source of capped transcripts for vmRNA transcription initiation. However, eRNAs account for only 3–5% of cap sources, whereas protein coding transcripts contribute over 70% [26]. Similarly, despite PROMPTs and mRNAs sharing the same bidirectional promoters, PROMPTs are used 12- to 40-fold less frequently. While transcript stability could also influence availability, RNA exosome inhibition experiments showed only modest effects on PROMPT usage [26], suggesting that transcript lifetime is not the primary determinant of cap source selection.

Instead, we hypothesize that if cap-snatching occurred immediately upon initiation while RNAPII was paused, the usage of sense and anti-sense transcripts would be roughly equivalent. The ability of FluPol to remain attached to the elongating RNAPII allows it to access the cap long after initiation, perhaps when steric hindrance between the transcription complex and the nascent RNA is reduced. This model aligns with evidence that cap-snatching occurs co-transcriptionally [26] and that capping enzymes maintain an association with the RNAPII CTD during transcript elongation and even beyond the site of polyadenylation ([29]). It is also noteworthy that pS5-RNAPII, which serves as an entry point for FluPol into gene bodies, persists during elongation, as this phosphorylation correlates with the co-transcriptional splicing process [71]. Furthermore, additional interactions with transcription machinery components, including elongation factors [13,22], are likely required for FluPol’s prolonged residence on transcribing genes, thereby enhancing cap-snatching opportunities.

### 4.3. FluPol Enrichment Across Host Defense Genes Concentrates Transcriptional Interference 

In this context, our data reveal that FluPol enrichment has a distinct pattern of associationat defense-related genes, while earlier genome-wide studies suggested that FluPol “passively” snatches caps from the most abundantly expressed transcripts [6]. The lack of correlation between FluPol recruitment and baseline expression levels demonstrates that FluPol does not simply target the most abundant transcripts in non-infected or infected cells (Figure A4E). Instead, there was a moderate yet significant correlation (rho = 0.22 − 0.40) with promoters marked by H3K27 acetylation upon infection—this epigenetic signature of active transcription suggests a preference for active promoters (Figure A4F), though the low determination coefficient (r^2^ ≈ 0.2) indicates that promoter activity alone does not fully explain its binding. A key observation is that FluPol enrichment is significantly stronger at promoters exhibiting infection-induced H3K27ac gain compared to overall promoters (Figure 3E). This suggests that IAV exploits the host’s transcriptional activation machinery while simultaneously attenuating gene expression, effectively decoupling activation marks from functional output. As the host cell rapidly activates defense genes during infection, FluPol’s presence at these newly opened promoters results in enrichment in genes of innate immune pathways. This pronounced accumulation across defense genes (evidenced by FluPol accumulation at TSSs (Figure 3F and Figure A4G–I), gene bodies (Figure 5G,H), TTSs (Figure 6D), and enhancers (Figure 4D) of immune-related pathways) represents a striking feature of host–virus interplay. This enrichment likely stems from interactions with infection-induced transcription factors or general transcriptional co-regulators [21,23,24,27]. While subtle sequence preferences of the PA endonuclease dictate the efficiency of cap-snatching on nascent host transcripts post-recruitment [7,28], promoter targeting itself is governed by host transcriptional activity and protein–protein interactions rather than sequence recognition by the PA catalytic site.

This sustained tracking with RNAPII thus serves a dual purpose, granting FluPol optimal co-transcriptional access for viral transcription while preferentially crippling the expression of host defense genes. Although the production of downstream-of-gene (DoG) RNAs is a known hallmark of cellular stress—including heat shock and endoplasmic reticulum (ER) stress [43,44]—the specific association of FluPol with these transcripts suggests more than a bystander effect. While stress-induced DoGs typically occur across a broad range of highly transcribed genes, our results demonstrate that FluPol-associated DoGs are specifically enriched for pathways involved in innate cellular defense. Crucially, as this enrichment is decoupled from overall transcriptional activity or H3K27ac abundance alone, it points toward a disproportionate association with host defense loci. By remaining associated with RNAPII through the termination site (Figure 6C) and within upregulated DoG regions (Figure 6B), FluPol may act as a local amplifier or stabilizer of termination defects at these key loci (Figure 6D). FluPol might physically hinder the recruitment or activity of termination factors, ensuring that these critical host messages are effectively suppressed. This model aligns with the observation that IAV-induced termination defects persist in the absence of NS1 [14], which suggests a multi-layered strategy where FluPol presence at active loci complements termination inhibition, ultimately contributing to the attenuation of the host’s most critical defense genes.

From an evolutionary standpoint, it is tempting to speculate that co-evolution has selected FluPol variants capable of preferentially exploiting or hijacking host transcriptional hubs specifically dedicated to antiviral defense. Whether this accumulation relies on a direct viral adaptor sensing immune-specific chromatin features or represents an optimized passive hitchhiking on host-driven RNAPII influx remains to be formally disentangled.

## Figures and Tables

**Figure 1:**
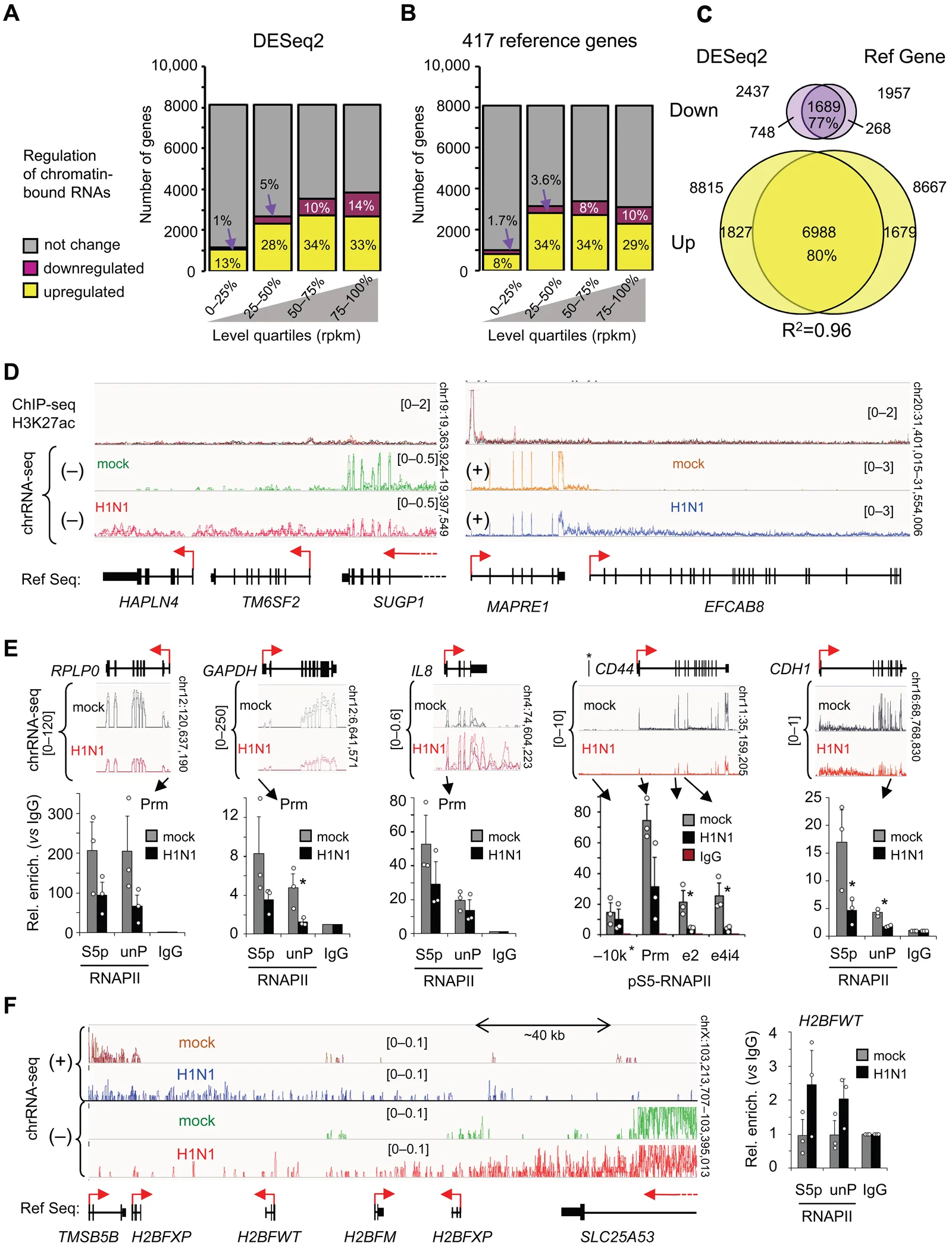
Chromatin-associated RNAs illustrate cell transcriptional changes induced by 6 h of IAV H1N1 infection in A549 cells. (**A**) Differential levels of chromatin-bound RNAs between H1N1-infected cells and uninfected (mock) cells were analyzed using DESeq2 package. Genes were considered regulated if the fold change was >1.5 and the adjusted *p*-value was <0.05. Genes were ranked in quartiles as indicated. (**B**) Differential RNA levels were analyzed by normalization based on the average variation of 417 reference genes that exhibited higher transcript levels (>300 rpkm) and long half-lives (>15 h) [56]. Genes were considered regulated if the fold change was >1.5 and the FDR was <0.05. Genes were ranked in quartiles from the mean of the two conditions. (**C**) Venn diagram showing the up- and downregulated genes evaluated by the two methods. R^2^ is the Pearson’s coefficient of determination comparing the two methods. (**D**) Examples illustrating the variation in read distribution in H1N1-infected cells from IGV visualization of indicated loci. The RNA-seq independent triplicates have been overlaid in the same color as indicated for each strand orientation indicated by (+) and (−). The top track shows H3K27ac enrichment on chromatin from infected or mock cells. Track scale ranges are indicated in brackets. The bottom track (RefSeq) shows the position and orientation of the genes as indicated by the red arrows. (**E**,**F**) ChIP assays using antibodies against S5-phosphorylated (S5p) (4H8 antibodies) or unphosphorylated (unP) (8WG16 antibodies) RNAPII from infected and uninfected cells. Immunoprecipitated DNA was quantified by qPCR using primers targeting the indicated regions. Enrichment is expressed relative to the signal obtained for ChIP with negative IgG control. Values are mean (±deviation) of three independent experiments. Statistical significance of differences between infected and uninfected cells was evaluated by Student’s *t*-test (two-tailed), with *p* < 0.05 (*) considered significant.

**Figure 2:**
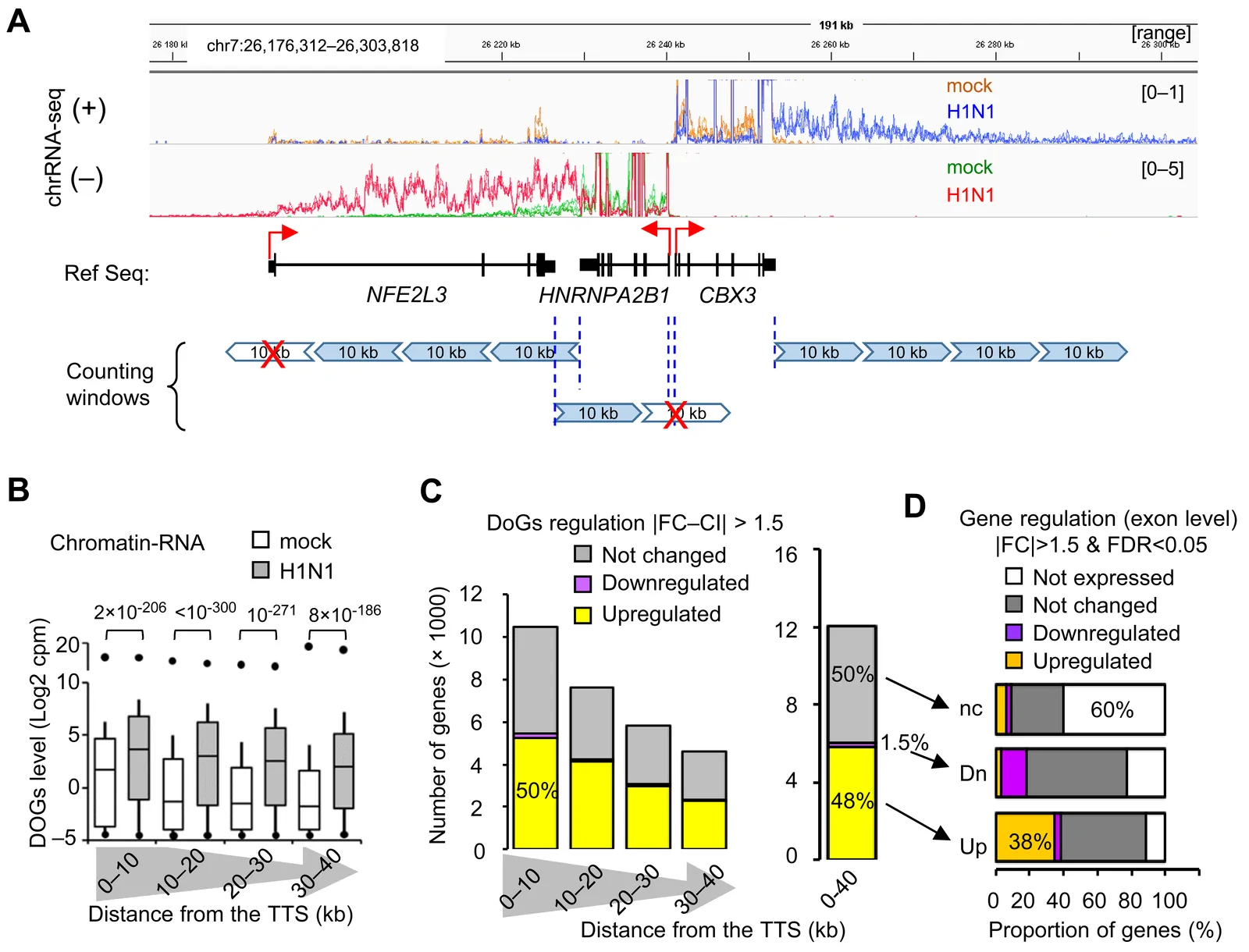
Chromatin RNAs were enriched in DoGs within the 3′ end region of the genes following IAV infection. (**A**) Strategy to quantify the 3′ end transcripts downstream of the termination sites (TTSs). Oriented reads were counted in 10 kb windows downstream of the TTS, extending up to 40 kb. The downstream 10 kb windows that overlapped with the transcription start site of another Ensembl gene in both orientations were excluded from the analysis (red crosses). (**B**) For each selected gene, the indicated 10 kb windows from the TTS were quantified for their DoG levels evaluated in log2 (cpm). Windows without reads were excluded from the analysis. Data are presented in box plots where the bottom whisker represents the 10th centile, the box corresponds to the 2nd and 3rd quartiles, the top whisker represents the 90th centile, and the middle line in the box denotes the median. Small circles indicate the minimum and maximum values. *p*-values shown at the top assess the statistical significance of the difference in DoG levels between infected and uninfected cells, calculated using a two-tailed Student’s *t*-test. (**C**) Number of genes exhibiting differential RNA levels within a 10 kb window downstream of the 3′ transcription termination site (TTS). DoG regions were classified as significantly altered when the fold change exceeded 50% relative to the uninfected cells (±90% confidence interval, CI). To prioritize transcriptionally active genes and minimize noise from transcriptional read-through, the analysis was restricted to genes within the top 75% of H3K27ac-enriched TSSs. (**D**) Proportional distribution of host gene regulation (as defined in Figure 1B) stratified by the status of the corresponding DoG regions (Upregulated [Up], Downregulated [Dn], or Unchanged [nc]).

**Figure 3:**
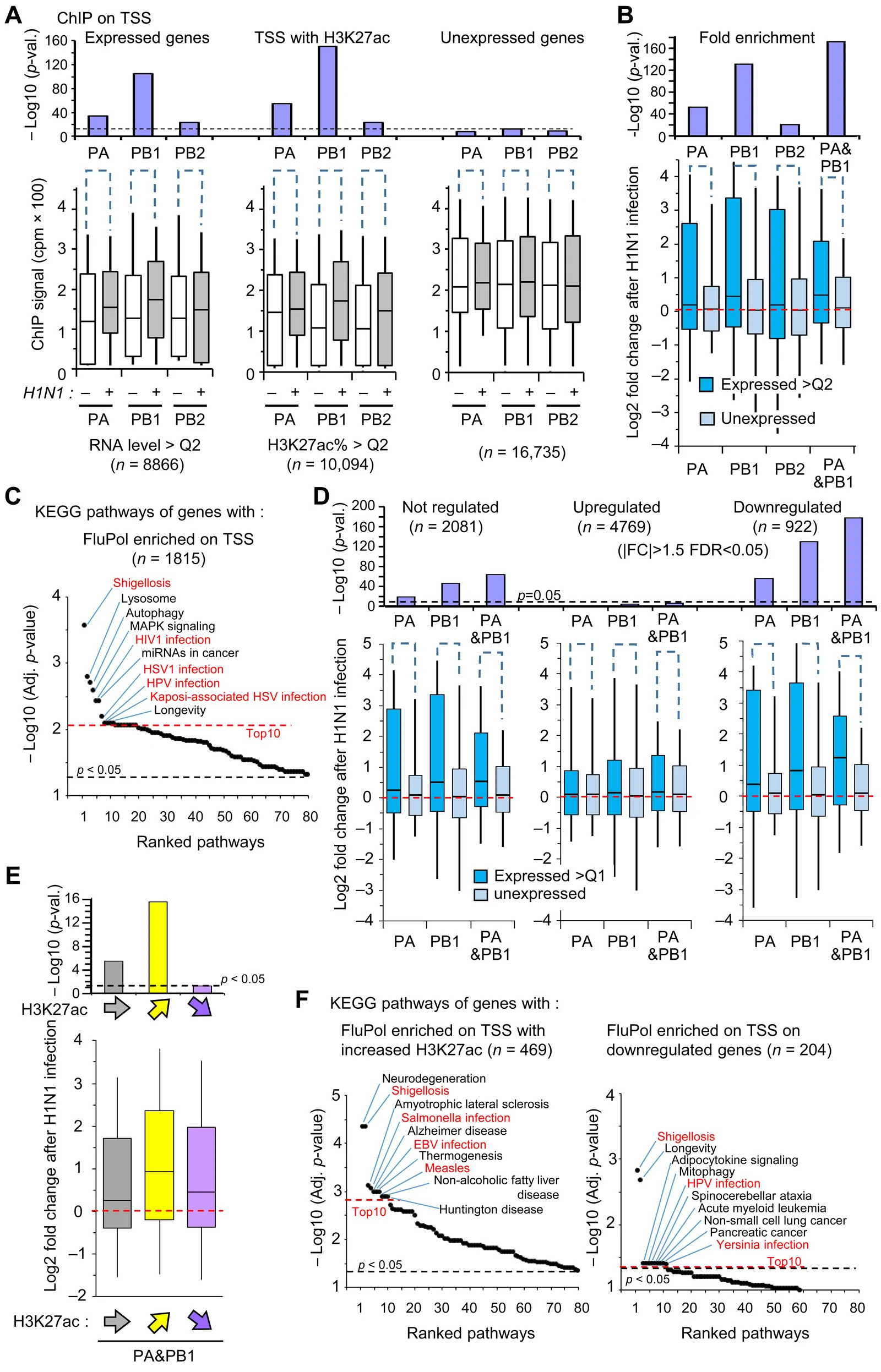
Enrichment of FluPol subunits on the chromatin. Chromatin from uninfected (−) or infected (+) A549 cells was immunoprecipitated with the indicated subunits of FluPol and analyzed by deep sequencing. For each 500 bp window centered on annotated transcription start sites (TSSs), DNA enrichment was calculated as the mean of replicates, expressed in cpm (multiplied by 100 for ease of presentation). Only TSSs separated from other TSSs by at least 5 kb were considered to avoid potential bias from closely spaced or overlapping genes. (**A**) Bottom box plots illustrate the distribution of TSSs from genes expressed above the median (>Q2), TSSs with H3K27ac signal greater than the median of H3K27ac signal, or TSSs from non-expressed genes. The bottom and top lines of the box plots represent the 10th and 90th percentiles, respectively. The number of TSSs tested (n) is indicated. The upper graphs show *p*-values, evaluated on log2-normalized cpm, using a paired two-tailed *t*-test to assess the enrichment induced by H1N1 infection for each TSS in the indicated categories. The dotted line represents the *p*-value threshold based on unexpressed genes. (**B**) The fold change in enrichment for the indicated TSS categories represents the average TSS ratio of infected versus uninfected cells for the FluPol subunits signal. Statistical analysis was conducted on log2 fold change using a two-tailed Student’s *t*-test to compare enrichment at TSSs of unexpressed genes (light blue) versus genes expressed above the median (blue). The dashed red line represents no change. The *p*-values are displayed above the box plots. Analyses of PA and PB1 were performed by combining their ChIP-seq signals (see methods). (**C**) KEGG pathways analysis performed on 1815 high-confidence FluPol-enriched genes. Enrichment was defined as significant PA and PB1 occupancy at the TSS (enrichment ≥ 50%, ±80% CI, see methods). The gene set was further filtered for high expression (top 75%) and active promoter marking (top 90% H3K27ac enrichment) to ensure the inclusion of active transcription units and the exclusion of read-through signals. Statistical significance was determined via Enrichr (adjusted *p*-values). The black dotted line indicates the 0.05 threshold; the red dotted line marks the top 10 enriched pathways. Red highlighting identifies infection-related pathways. (**D**) Fold change in enrichment for TSSs of genes in the indicated categories. As described in (**B**), enrichment of the indicated FluPol subunits was analyzed on the TSSs of the top 75% most highly expressed genes, either regulated by more than 50% (FDR < 0.05) or not regulated. Enrichments were compared to the FluPol subunit signal at TSSs of unexpressed genes using a two-tailed Student’s *t*-test to calculate *p*-values. (**E**) Fold change in enrichment, as described in (**B**), for PA and PB1 signals on TSSs, classified according to H3K27ac differential levels upon infection: unchanged (gray, *n* = 8727), increased (yellow, *n* = 2353) or decreased (violet, *n* = 2437). H3K27ac levels were normalized to H3 levels and considered regulated if, among the top 90% most enriched TSSs in H3K27ac, the change exceeded 20% (±90% CI). These enrichments were compared to all TSSs with equivalent H3K27ac levels to calculate *p*-values using a two-tailed Student’s *t*-test. (**F**) KEGG pathway analysis of FluPol-targeted genes (enrichment ≥ 50%, ±80% CI, for PA and PB1) stratified by H3K27ac dynamics and RNA-level regulation. Analysis was performed as described in (**E**) for genes exhibiting increased H3K27ac levels (≥20% ± CI, *n* = 469) or exhibiting downregulated RNA level (≥50%, FDR < 0.05, *n* = 204). All the gene lists are available in Table S3.

**Figure 4:**
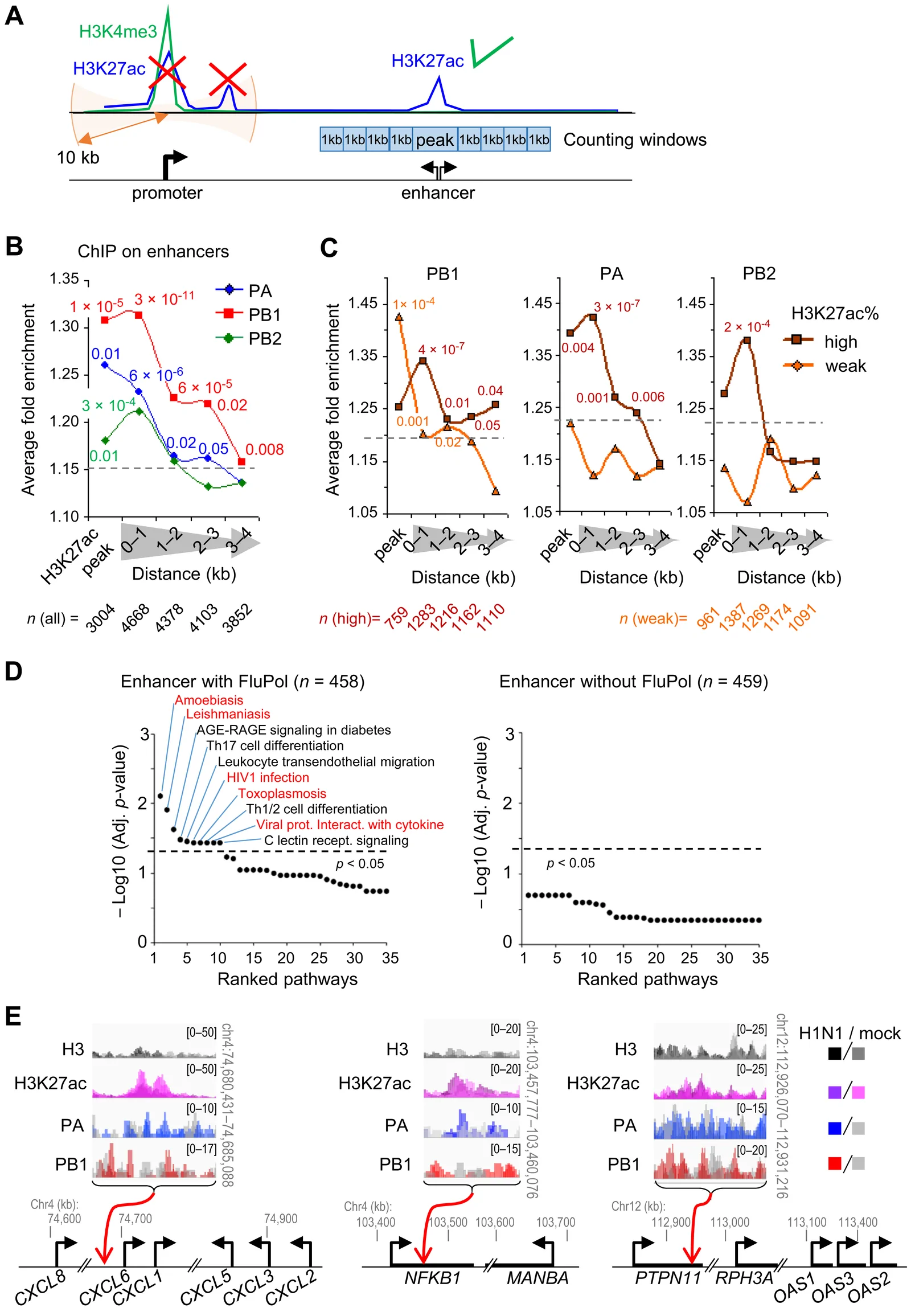
FluPol subunits on chromatin are enriched at the enhancer sites. (**A**) Strategy to quantify FluPol enrichment around H3K27ac peaks defining enhancers. Reads were counted within the H3K27ac window and in four 1 kb windows both downstream and upstream of the boundaries of the H3K27ac peaks. For each counting window, the fold change in enrichment of the indicated FluPol subunits between infected cells and mock cells was calculated. H3K27ac peaks overlapping the 10 kb region surrounding H3K4me3 peaks were excluded from the analysis (indicated by red crosses). (**B**) Peaks of H3K27ac detected in infected cells that did not overlap with H3K4me3 peaks (±10 kb) defined the first counting windows (referred to as “peak”). Upstream and downstream windows were pooled for each indicated distance. The average enrichment ratio of the indicated FluPol subunits between infected cells and mock cells is presented. (**C**) The same calculation was performed for enhancers with high or low percentages of H3K27ac relative to H3. Enhancer peaks were divided into quartiles based on H3K27ac%, and the relative enrichment of FluPol subunits is shown for the 4th quartile (high level in brown) and for the 2nd quartile (weak level in orange). (**B**,**C**) Differences between infected and mock cells were evaluated using a paired *t*-test, and significant *p*-values are indicated. The number of loci analyzed is shown above the graphs. The height of the dashed lines represents the fold enrichment threshold at which differences are considered significant. (**D**) Functional enrichment analysis of genes associated with FluPol-occupied enhancers. KEGG pathway analysis was performed on genes located within 1 Mbp of H3K27ac peaks (top 90th percentile of H3K27ac content). (**Left**) Enrichment for 458 enhancers showing high FluPol occupancy (PA and PB1 ChIP-seq signal ≥ 50% above background (with 80% CI) within the peak or ±1 kb flanking windows), associated with 3511 expressed genes (>Q1 RNA level; Table S4A). (**Right**) Enrichment for 459 control enhancers with comparable H3K27ac levels but minimal FluPol occupancy (PA and PB1 signal < 5%), associated with 3947 expressed genes (Table S4B). For each peak, the 20 nearest genes were filtered for expression (>Q1) prior to analysis. Pathways are ranked by adjusted *p*-value; the dotted line indicates the threshold for statistical significance (*p* < 0.05). (**E**) Localization of enhancers enriched by FluPol. Examples of enhancers and their neighboring genes are shown to illustrate the pathways in (**D**). ChIP-seq independent replicates performed with antibodies against the indicated proteins in infected or mock cells are overlaid in the specified colors. Black arrows indicate the positions of the TSSs and the orientation of the gene, while red arrows mark the locations of the enhancer regions where FluPol enrichment was detected. The y-axis scale for each track is indicated in brackets.

**Figure 6:**
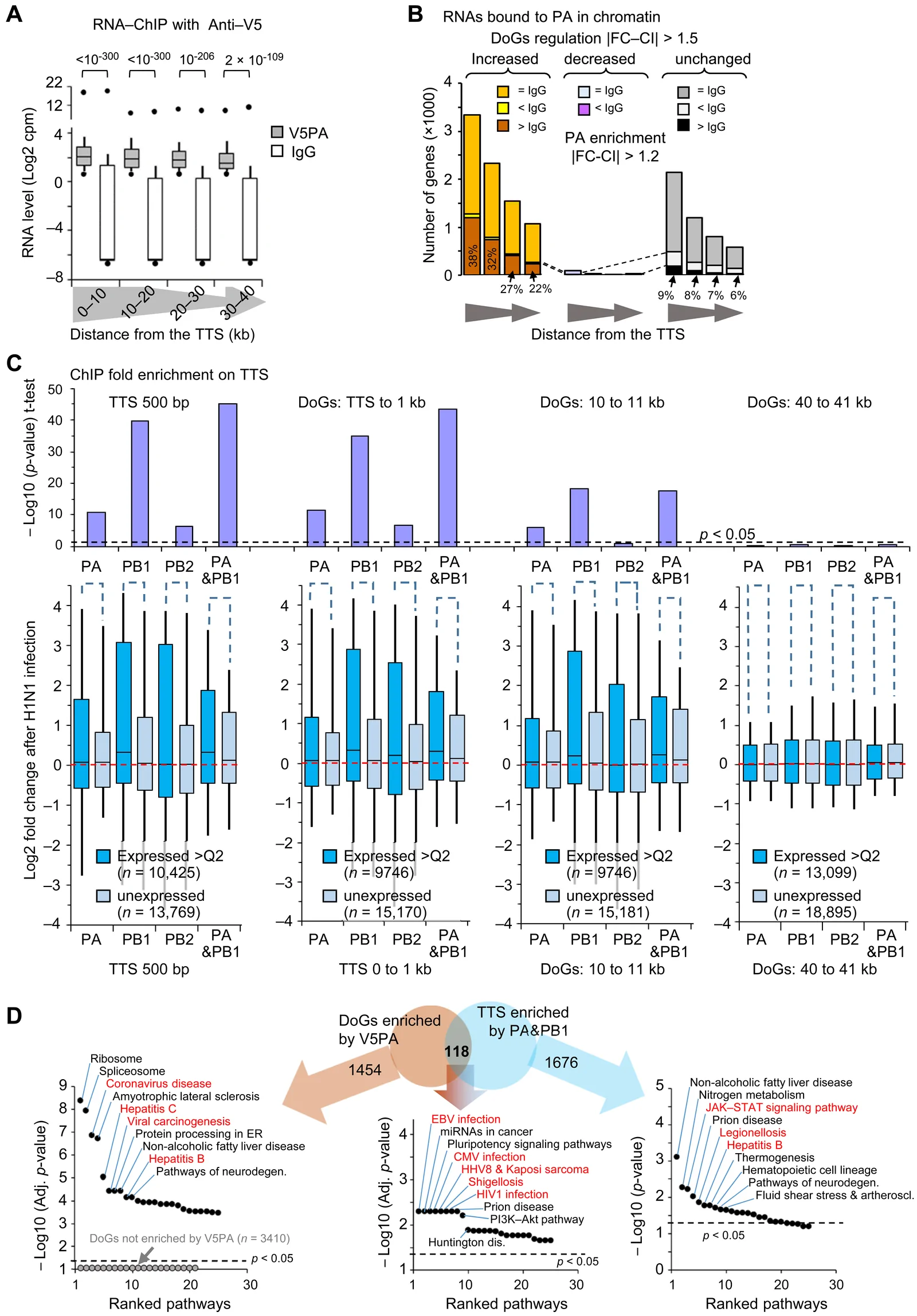
Enrichment of FluPol subunits on chromatin downstream of the 3′ ends of genes. (**A**) Chromatin-associated RNA from infected A549 cells was immunoprecipitated using the indicated antibodies and analyzed by deep sequencing. For each selected gene, DoG levels in 10 kb windows downstream of the transcription termination site (TTS) were quantified and expressed as log2(cpm). Only windows containing ≥5 reads that did not overlap with neighboring genes were included in the analysis. Data are presented as box plots, formatted similarly to Figure 2B. (**B**) Counts of V5-PA-enriched DoG regions (brown and black) and their differential RNA levels upon infection. Significant regions were defined by an absolute difference ∣V5-PA − IgG∣ > 1.2, (90% CI). DoG RNA levels were considered significantly modified if the lower bound of the 95% CI for the fold change was greater than 1.5. The percentages of DoG region enriched in PA ChIP are indicated. (**C**) Fold change in enrichment for categories of the indicated terminal regions relative to TTS. These values represent the average differences in log2 cpm between infected and uninfected cells. Statistical analysis was conducted on log2 fold change using a two-tailed Student’s *t*-test to compare DoG regions from unexpressed genes (light blue) and genes expressed above the median (blue). The dashed red line represents no change. *p*-values are displayed above the box plots, with the dashed line marking the threshold of *p* < 0.05. The number (n) of TTS tested is indicated for each category (see Section 2). (**D**) Pathway analysis of genes with enriched DoG in RNA-ChIP with PA-V5 and in TTS regions in ChIP for PA and PB1; 1454 genes were identified as enriched in RNA-ChIP V5-PA analysis, with at least a 1.2-fold increase (with 90%CI) in the first 10 kb windows downstream of the TTS (left panel). A total of 1676 genes were selected from expressed genes for ChIP analysis to have the 1 kb windows downstream of the TTS enriched at least 1.5-fold (with 80% CI) for PA and PB1 in infected versus control cells (right panel); 3410 genes without V5-PA-enriched DoGs exhibit no significant pathway (gray plots). Additionally, 118 common genes from the two independent approaches were analyzed (middle panel). For each gene list, regulation pathways were analyzed using Enrichr, and the first 10 most significant KEGG pathways are shown with adjusted *p*-values for V5-PA RNA-ChIP-seq and common genes and non-adjusted *p*-values for FluPol ChIP-seq on TTS. The dotted line marks the threshold of *p* < 0.05.

**Figure 7:**
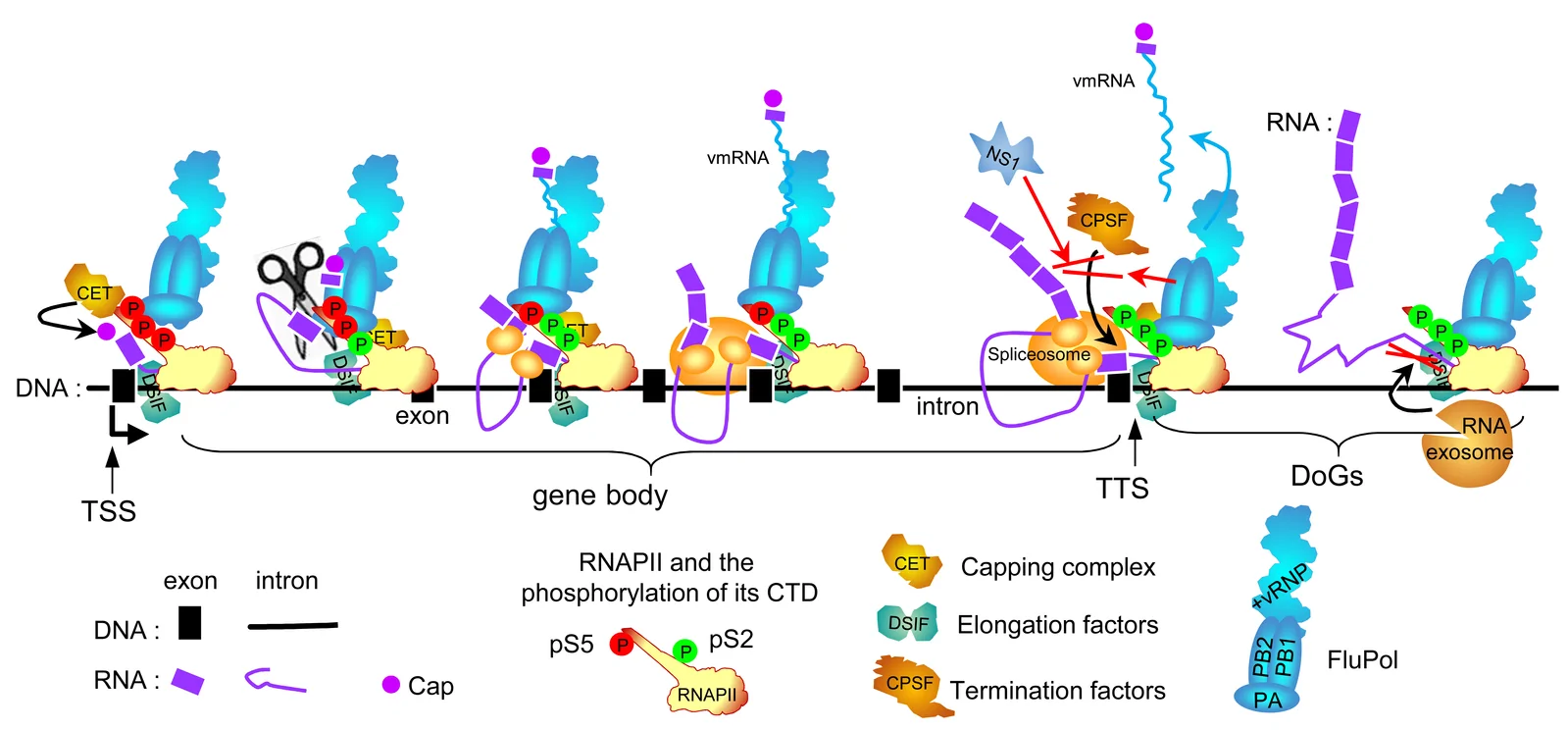
Working model of the functional association of FluPol and RNAPII. FluPol is initially recruited to transcription start sites (TSSs), where the PB2 subunit binds to the cap of nascent RNA. During transcription elongation, FluPol remains associated with RNAPII through interactions with the serine-5-phosphorylated (S5p) C-terminal domain (CTD) of RNAPII, which persists throughout elongation. Additional interactions with elongation factors or the nascent transcript itself may contribute to this stable association. Concurrently, co-transcriptional splicing of the nascent transcript generates intermediate spliced forms that remain associated with FluPol via RNAPII. At the 3′ end of genes, FluPol may inhibit the termination complex, including cleavage and polyadenylation specificity factor (CPSF), which is also targeted by NS1. This inhibition, along with suppression of the RNA exosome, promotes the stabilization of downstream-of-gene transcripts (DoGs). The PA subunit of FluPol (depicted as “scissors”) may perform cap-snatching reactions on nascent RNA at any stage of this process. The nascent RNA remains bound to RNAPII throughout elongation and into the 3′ end region, where RNAPII persists beyond the transcription termination site (TTS) due to impaired termination. This model accounts for the observed enrichment of FluPol on DoGs and chromatin-associated exonic RNAs.

**Figure A1:**
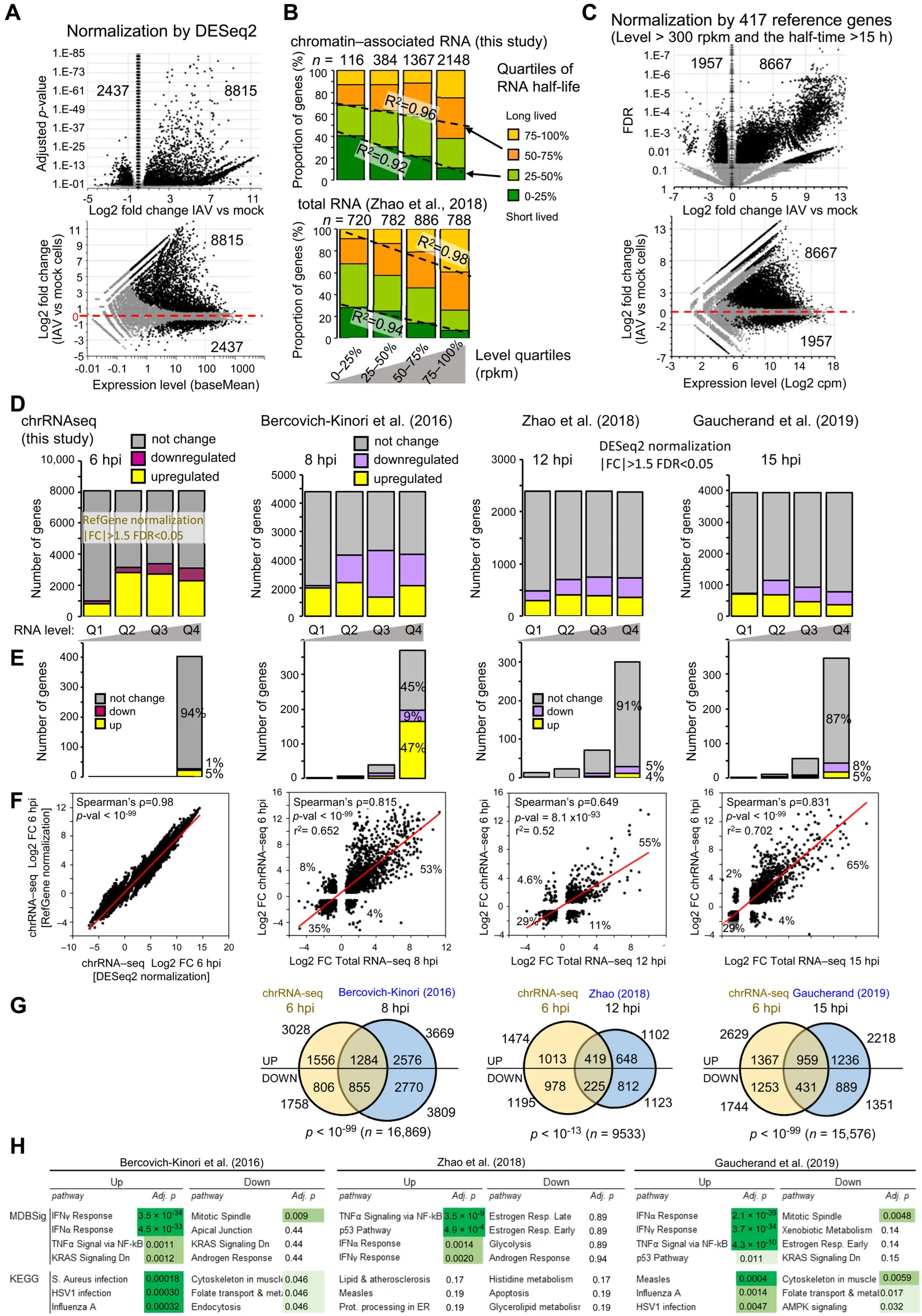

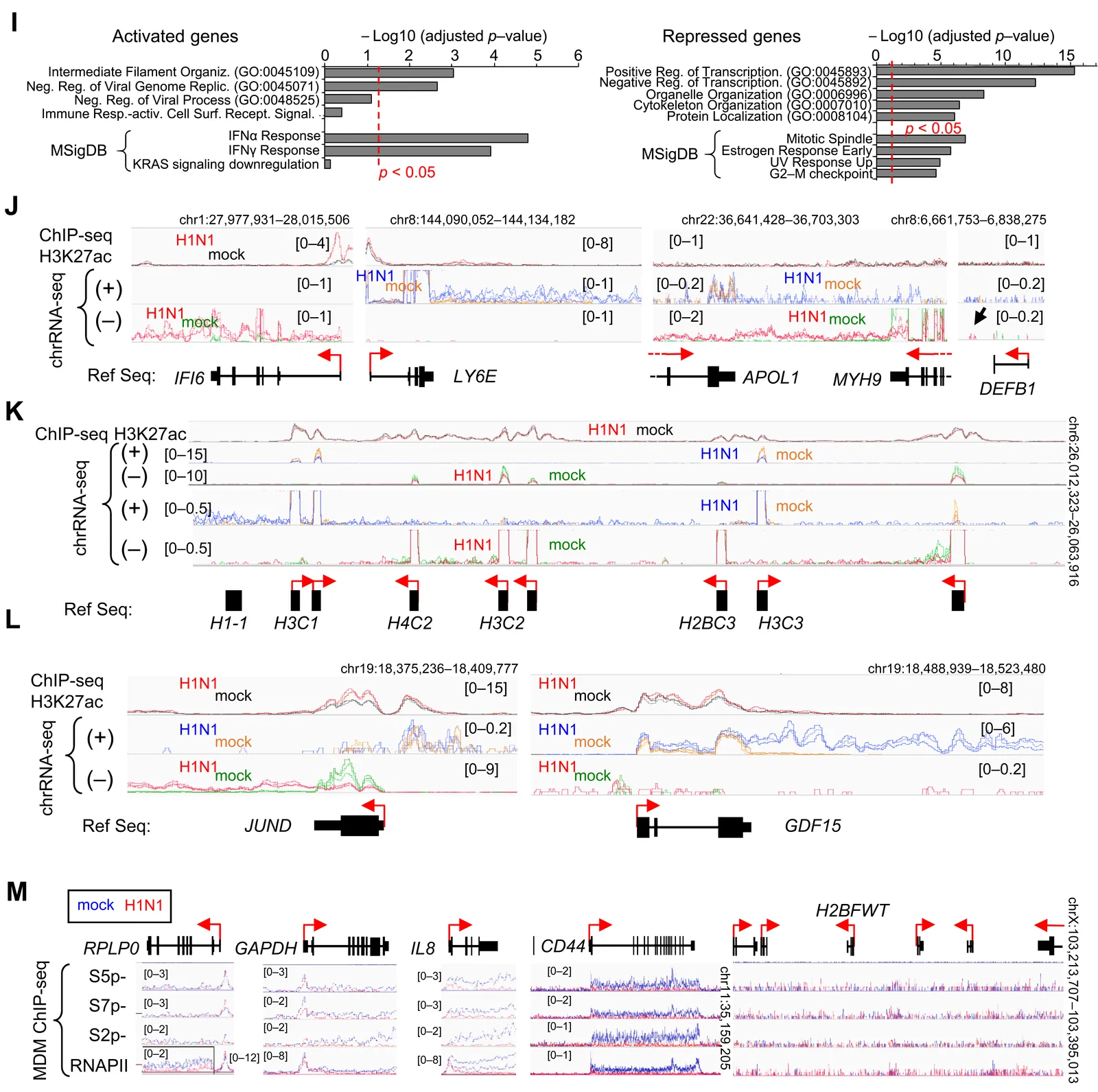
(**A**) Differential RNA levels between H1N1-infected and uninfected cells using DESeq2 package. Variations were considered significant (black dot) if the mean fold change of IAV versus mock cells (FC) was >1.5 and the adjusted *p*-value < 0.05. The number of black dots is indicated. (**B**) Validation of the reference gene set for chrRNA-seq normalization (RefGene). Proportion of genes stratified by expression level in A549 chromatin-associated RNA in mock and infected cells at 6 hpi (top; *n* = 4015 genes detected out of 4678 from [56]) or total RNA in mock and 12 hpi infected cells (bottom; *n* = 3176 genes from [40]), relative to their mRNA half-life quartiles. Pearson’s coefficient of determination (R^2^) was calculated between the maximum expression level (rpkm) observed between mock and infected conditions and the percentage of genes in each half-life quartile (indicated by dashed lines and arrows). (**C**) Differential levels of chromatin-associated RNA using normalization based on the average variation of 417 reference genes expressing higher levels (>10 log2 cpm) of long half-life (>15 h) transcripts [56]. Variations were considered significant (black dot) if the mean fold change of IAV versus mock cells was >1.5 and the FDR < 0.05 as calculated by paired *t*-test (two-tailed) on log2(cpm). (**A**,**C**) Note that visible striping at lower signal intensities (MA plots) or lower significance levels (Volcano plots) reflects discrete integer count values among low-abundance transcripts included in the broad dataset. (**D**) Comparison of differential gene expression between our chrRNA-seq and total RNA-seq across datasets [37,38,40]. Datasets were analyzed using DESeq2, and infection time points showing significant changes were used for subsequent comparisons. (**E**) Stability of the 417 reference genes (highly expressed, half-life > 15 h) across datasets. Reference genes identified from our 6 hpi chrRNA-seq dataset were plotted by expression level quartile for each study, and the proportion of differentially expressed genes (|FC| > 1.5, FDR < 0.05) is indicated. Approximately 90% of these genes remain stable and highly expressed in external datasets. (**F**) Correlation of fold changes between our 6 hpi chrRNA-seq dataset and external total RNA-seq datasets. The left panel shows a scatter plot comparing DESeq2 and RefGene normalization for our chrRNA-seq data. The right panels show comparisons between our RefGene-normalized 6 hpi chrRNA-seq dataset and the indicated external datasets normalized by DESeq2. Spearman’s rho, the corresponding *p*-value, and Pearson’s coefficient of determination (r^2^) are indicated for each correlation. (**G**) Overlap of upregulated and downregulated genes between our dataset and external studies. The number of shared genes is shown along with *p*-values from exact hypergeometric tests (Fisher’s exact test) evaluating whether the overlap is significantly different from random expectation. (**H**) Pathway enrichment analysis of overlapping genes evaluated via Enrichr. The top molecular signatures and KEGG pathways are shown with their adjusted *p*-values. Colored *p*-values indicate *p* < 0.05, with increasing intensity for *p* < 0.01 and *p* < 0.001. Upregulated genes are enriched in antiviral pathways, whereas downregulated genes are associated with cytoskeleton organization and folate metabolism. (**I**) Pathway analysis of the 6928 genes upregulated 4-fold with FDR < 0.05 between infected and mock cells (left panel), and the same analysis of the 1957 genes downregulated 1.5-fold with FDR < 0.05 (right panel). Adjusted *p*-values of Gene Ontology (GO) 2025 and Molecular Signature DB 2020 were evaluated by Enrichr, and the threshold of *p* < 0.05 is indicated by the dotted red line. (**J**–**L**) Examples illustrating the variation in read distribution in H1N1 infected cells from IGV visualization of indicated loci. The RNA-seq independent triplicates have been overlaid in the same color as indicated for each strand orientation. The top trace shows the enrichment of H3K27ac on the chromatin of infected or mock cells. The scale range of the tracks is indicated in parentheses. The bottom track (RefSeq) shows the position of the genes and their orientation as indicated by the red arrows. (**M**) Distribution of indicated phosphorylated (at serine-5, 7 and 2) and total RNAPII on analyzed genes (Figure 1E). ChIP-seq assays using antibodies against the indicated RNAPII were performed on infected (red line) or uninfected MDM cells (blue line) from the GSE103477 dataset [41]. The range scale is indicated in brackets.

**Figure A2:**
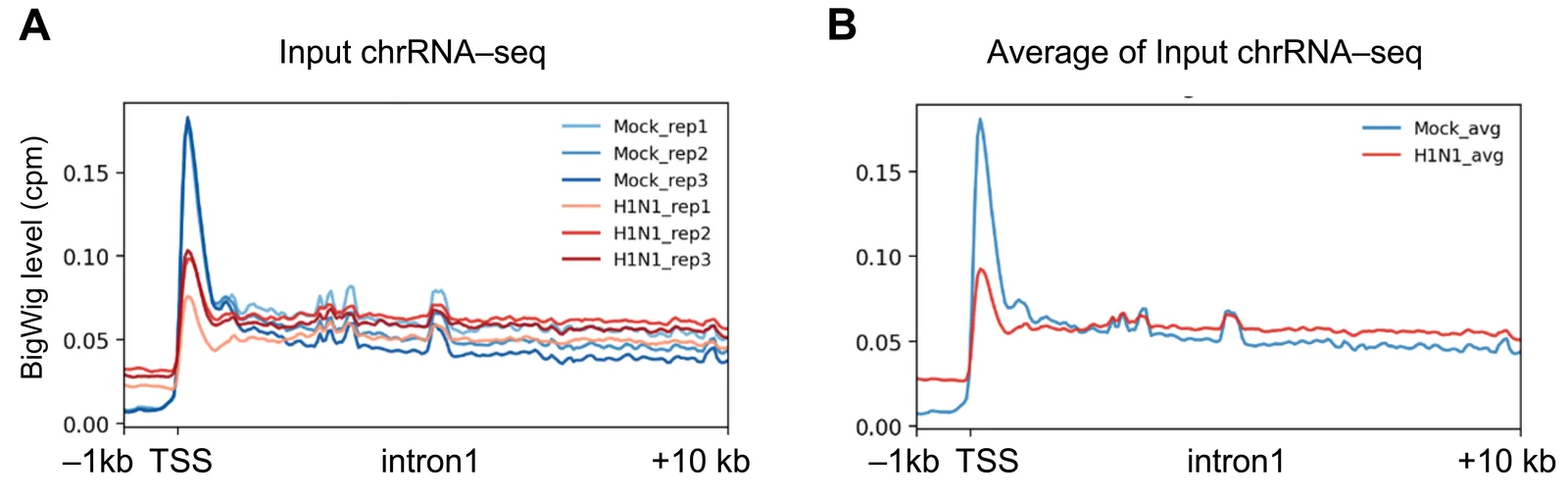
Metagene profiles of chromatin-associated RNA (chrRNA-seq) signal from −1 kb upstream of the TSS to +10 kb downstream of the TSS for 2288 expressed genes containing first introns > 10 kb.(**A**) Individual profiles for the three biological replicates in mock-treated (blue) and H1N1-infected (red) A549 cells. (**B**) Average metagene profile across the three replicates for mock (blue) and H1N1-infected (red) conditions. Metagene profiles were generated using cpm-normalized BigWig files.

**Figure A3:**
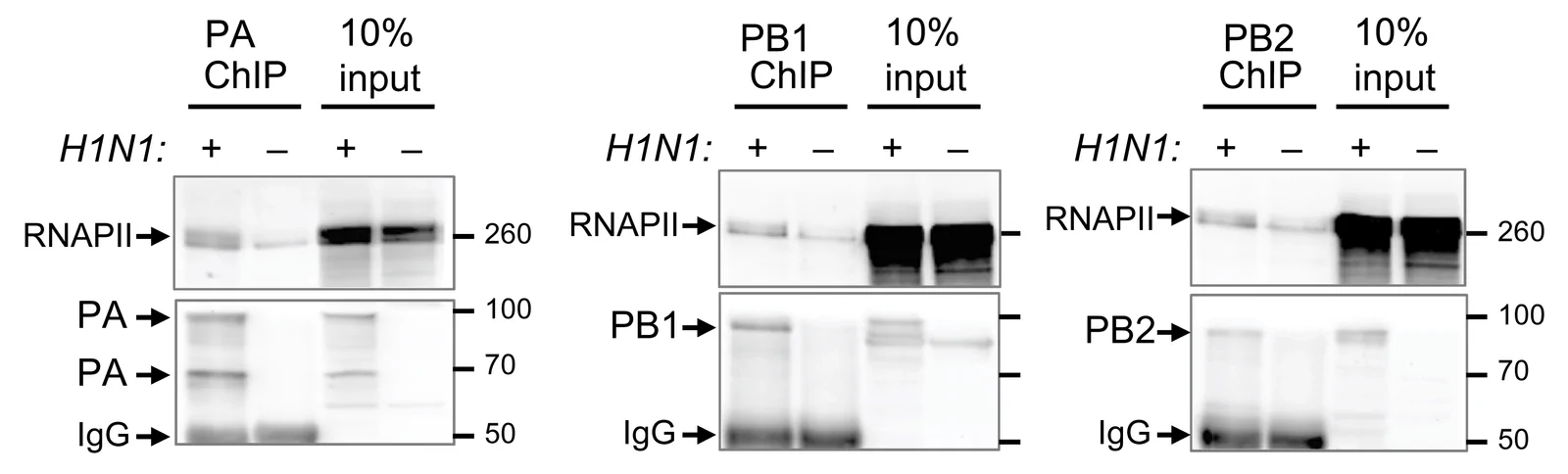
Whole-cell extracts were prepared from infected (+) or uninfected (−) cells and subjected to immunoprecipitation with the indicated antibodies used in the ChIP assay. The eluates and 10% of the extracts (input) were resolved by Western blot. The lower part of the membranes (50 to 120 kDa) was detected with the corresponding antibodies and the upper part (120 kDa-top) with pS5 RNAPII antibodies.

**Figure A4:**
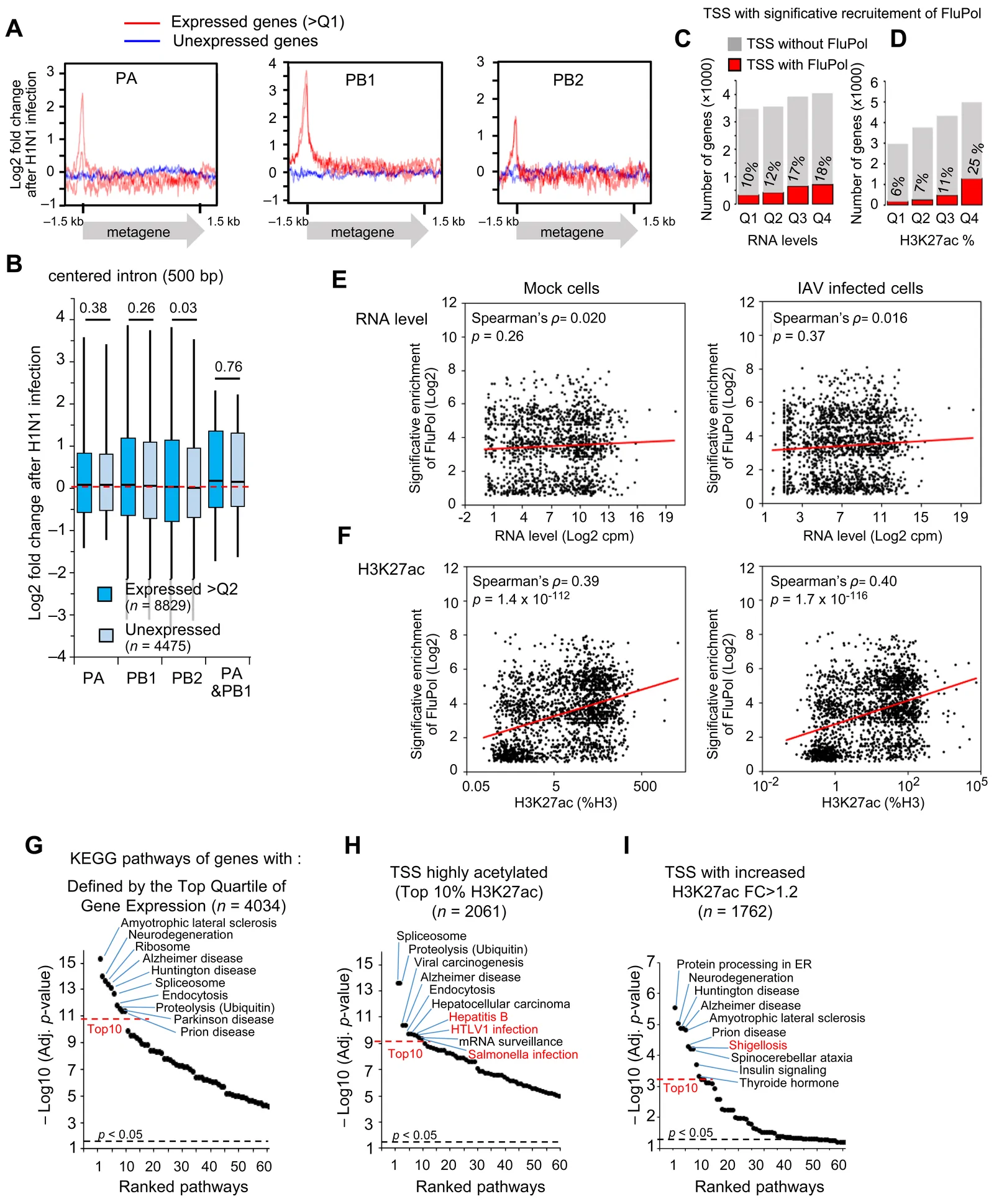

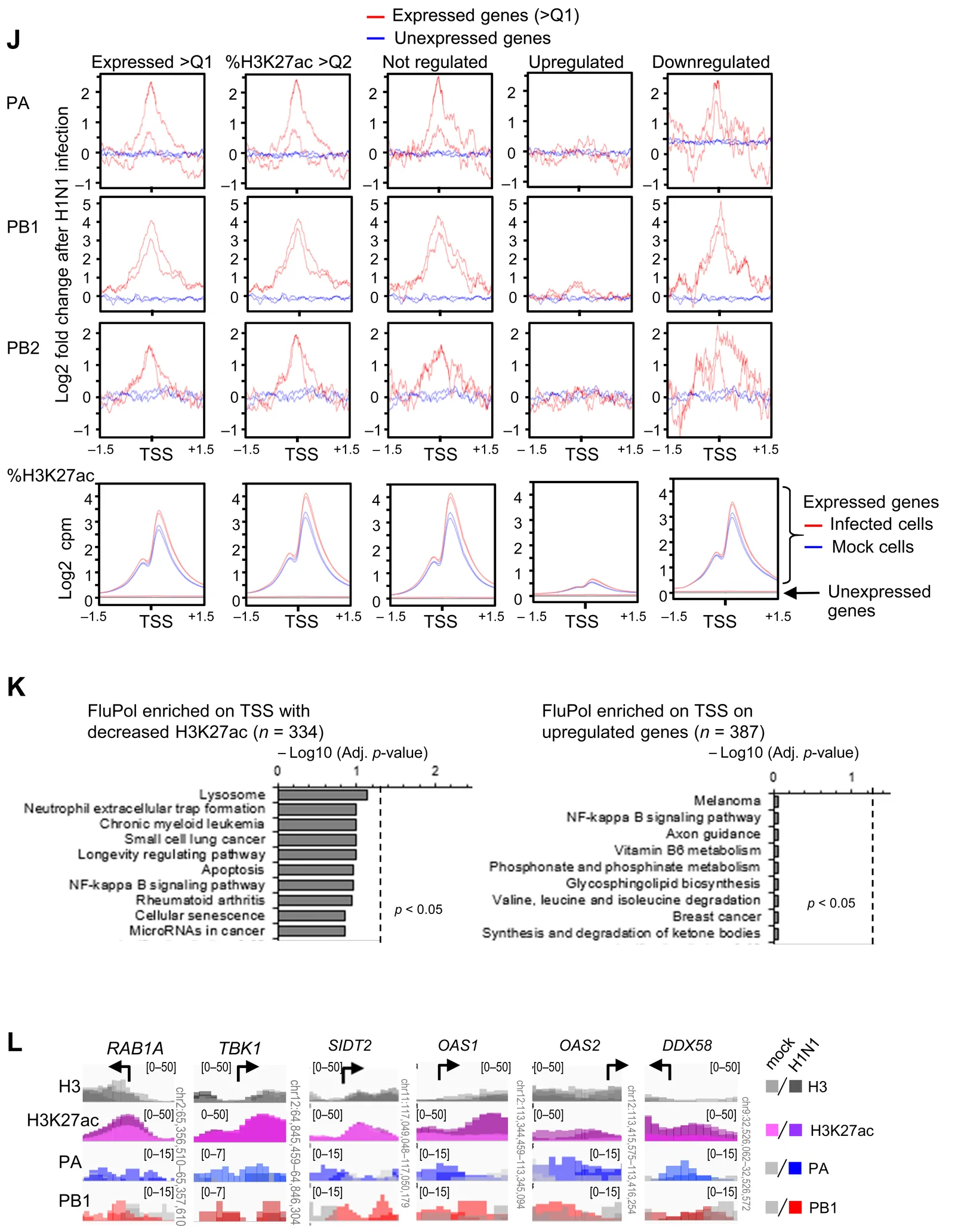
(**A**) Profiles covering entire gene bodies including introns (metagenes) of FluPol subunits signals at 7879 expressed genes (with rpkm > Q1 and with H3K27ac signal over the median) to ensure the inclusion of bona fide transcriptionally active genes and the exclusion of potential RNA signals originating from transcriptional readthrough. ChIP-Seq signals from expressed genes (in red) are compared to those at 16,735 unexpressed genes (in blue). Coverages are expressed as fold change between infected and mock cells. (**B**) The same analysis, as in Figure 3B, was performed for the averages of intronic coverage. For each gene, the more central intron was used to define centered 500 bp counting windows. Only windows separated by 10 kb from the TSS were considered. (**C**) Distribution of FluPol recruitment at TSSs across quartiles of chromatin-bound RNA levels. The bar plot shows the percentage of TSSs isolated (≥5 kb from neighboring TSSs) and enriched for H3K27ac signal (top 90th percentile, gray portion). The 1815 genes with significant FluPol recruitment, as defined in Figure 3C, are indicated in the red portion of the bars. Genes were categorized by quartiles of chromatin-bound RNA levels, as defined in Figure 1B. Percentages indicate the proportion of TSSs with significant FluPol enrichment in each RNA quartile. (**D**) Distribution of FluPol recruitment at TSSs across quartiles of H3K27ac enrichment. The bar plot shows the percentage of TSSs isolated (≥5 kb from neighboring TSSs) and with exonic RNA coverage exceeding the minimum CPM threshold. The 1815 genes with significant FluPol recruitment (as defined in Figure 3C) are highlighted in red. Genes were stratified by quartiles of H3K27ac enrichment relative to H3 signal. Percentages indicate the proportion of TSSs with significant FluPol enrichment in each H3K27ac quartile. (**E**,**F**) Scatter plots showing the correlation between FluPol ChIP signal (evaluated by the combined PA and PB1 ChIP-seq signal; see Section 2) and either chromatin-associated RNA levels in non-infected ((**E**), left) and infected cells ((**E**), right) or H3K27ac levels in non-infected ((**F**), left) and infected cells ((**F**), right). Correlations were evaluated considering only 3007 genes with significant FluPol enrichment (|FC ± 80% CI| > 1.5). Spearman’s rho and corresponding *p*-values are indicated for each correlation. This difference in correlation between FluPol and either RNA levels or H3K27ac is conserved when considering the whole FluPol ChIP-seq signal: correlation with RNA is very weak (non-infected: Spearman’s rho = 0.0133, *p* = 0.014; infected: Spearman’s rho = 0.0092, *p* = 0.095), whereas correlation with H3K27ac levels at promoters is stronger (non-infected: Spearman’s rho = 0.222, *p* = 4.9 × 10^−274^; infected: Spearman’s rho = 0.224, *p* = 4.9 × 10^−277^). (**G**–**I**) KEGG pathway (2021) profiling of highly expressed genes and highly H3K27ac-enriched gene for comparison with analysis of FluPol-enriched genes (Figure 3C). Enrichment analysis was performed on genes exhibiting the top 25% of transcript abundance (*n* = 4034; (**G**)), the top 10% of H3K27ac signal (*n* = 2061; (**H**)), or TSS with increased H3K27ac by 1.2-fold (80% CI) (*n* = 1762; (**I**)). Adjusted *p*-values were calculated via Enrichr. Significance thresholds (*p* < 0.05) and the top 10 ranking pathways are indicated by black and red dotted lines, respectively. The full lists of pathways and related genes are in Table S3. (**J**) Profiles of FluPol subunit signals covering the TSS region (from −1.5 kb to +1.5 kb) are shown for expressed genes (as defined in Figure A1B, *n* = 7879), for genes with TSS regions with H3K27ac levels higher than the median (*n* = 6626), and for unregulated (2081), upregulated (*n* = 4769), and downregulated (*n* = 922) genes (as defined in Figure 3D), comparing infected and mock cells (red lines). Each graph includes reference profiles for 16,735 unexpressed genes (blue lines). The coverages are expressed as fold changes between infected and mock cells. As a control, profiles of H3K27ac enrichment in infected cells (red) and mock cells (blue) are displayed at the bottom. (**K**) The same KEGG pathway 2021 analysis conducted in Figure 3F was performed on FluPol-targeted genes exhibiting decreased H3K27ac levels (≥20% ± IC, *n* = 334) (left) or exhibiting upregulated RNA level (≥50% ± IC, *n* = 387) (right). (**L**) Examples of 500 bp regions around the TSS of the indicated genes (selected in the pathways in Figure 3C). The ChIP-seq independent replicates performed with antibodies against the indicated proteins in infected or mock cells are overlaid in the indicated color. The black arrows indicate the position of the TSS and the orientation of the gene. The *y*-axis scale for each track is indicated in brackets.

**Figure A5:**
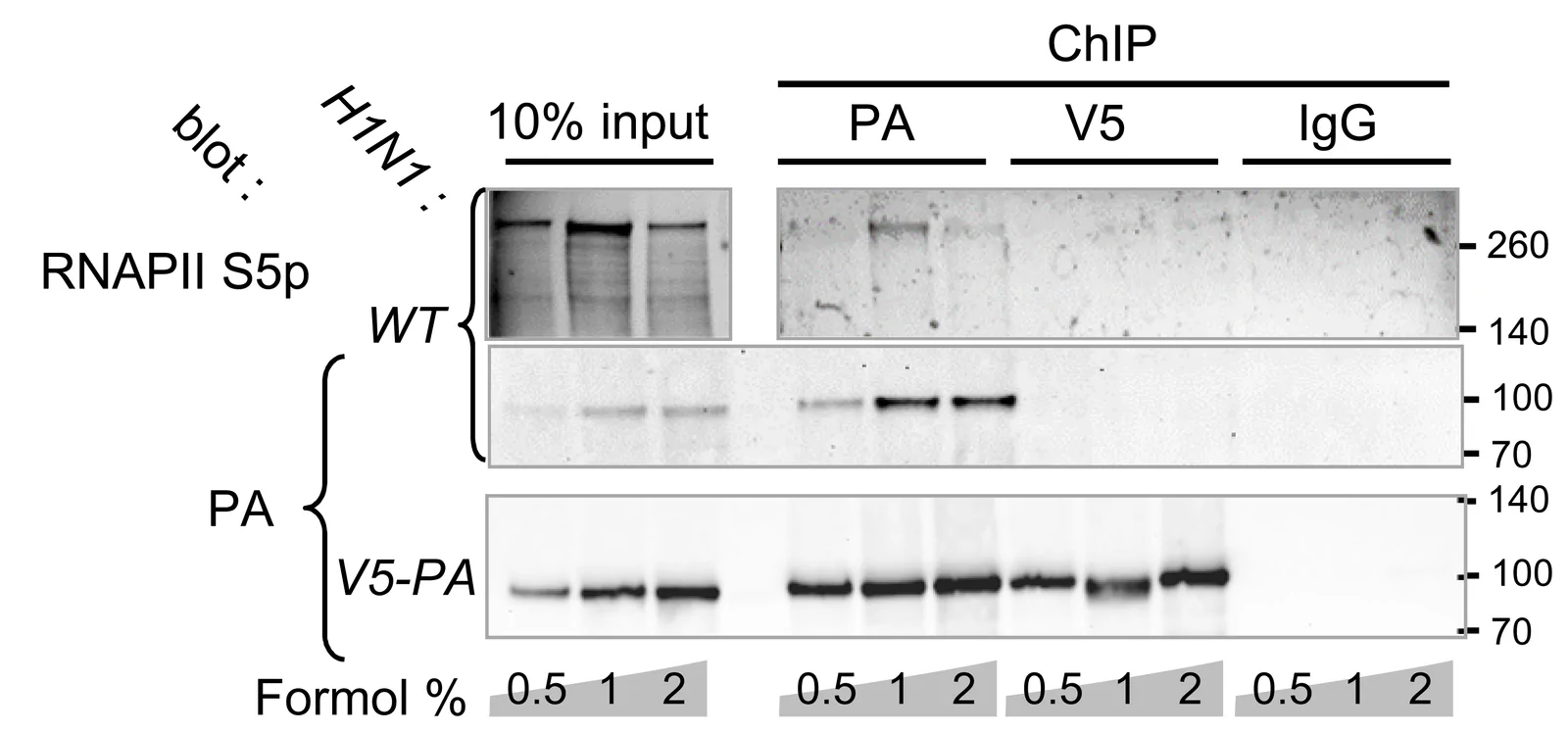
A549 cells infected by WT H1N1 or V5-PA-engineered H1N1 crosslinked with the indicated concentration of formaldehyde or left uncrosslinked. Chromatin fractions were subjected to immunoprecipitation with PA, V5 antibodies or non-immune IgG. Eluates and 10% of extracts were resolved by Western blot. The lower part of the membranes was visualized with PA antibodies and the upper part with phospho-RNAPII antibodies.

**Figure A6:**
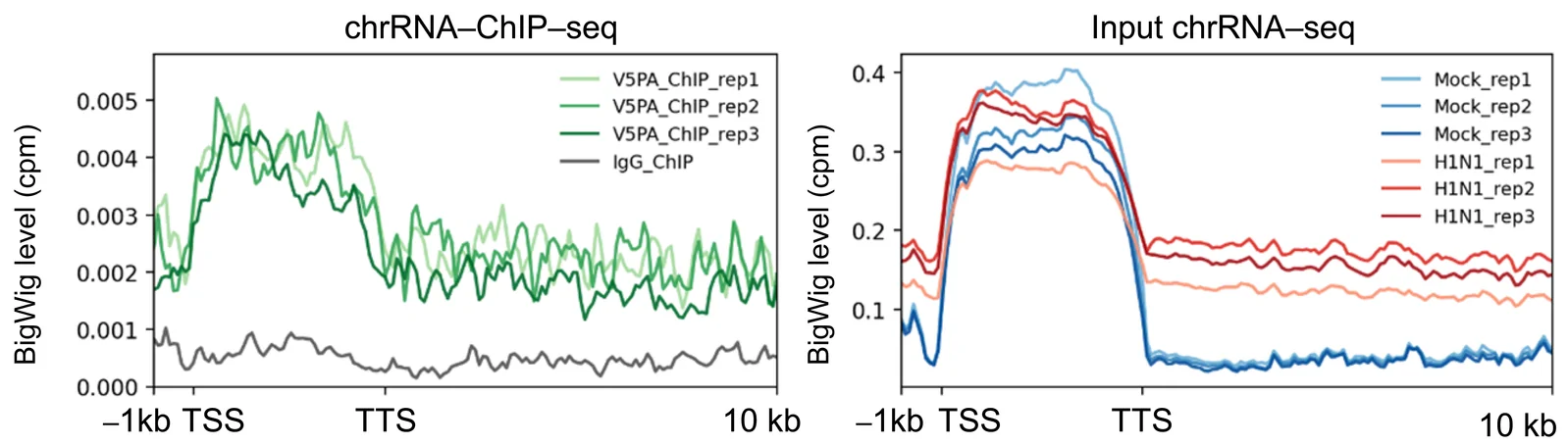
Normalized coverage profiles (cpm) were generated from BigWig files across the gene body and 10 kb region downstream of the TTS for 1123 selected genes, filtered to remove repetitive elements that cause signal noise. Left panel shows V5-PA chrRNA-ChIP-seq signal (green) compared to IgG control (gray). Right panel displays corresponding input chrRNA-seq coverage in virus-infected (red) and mock-infected (blue) cells.

**Figure A7:**
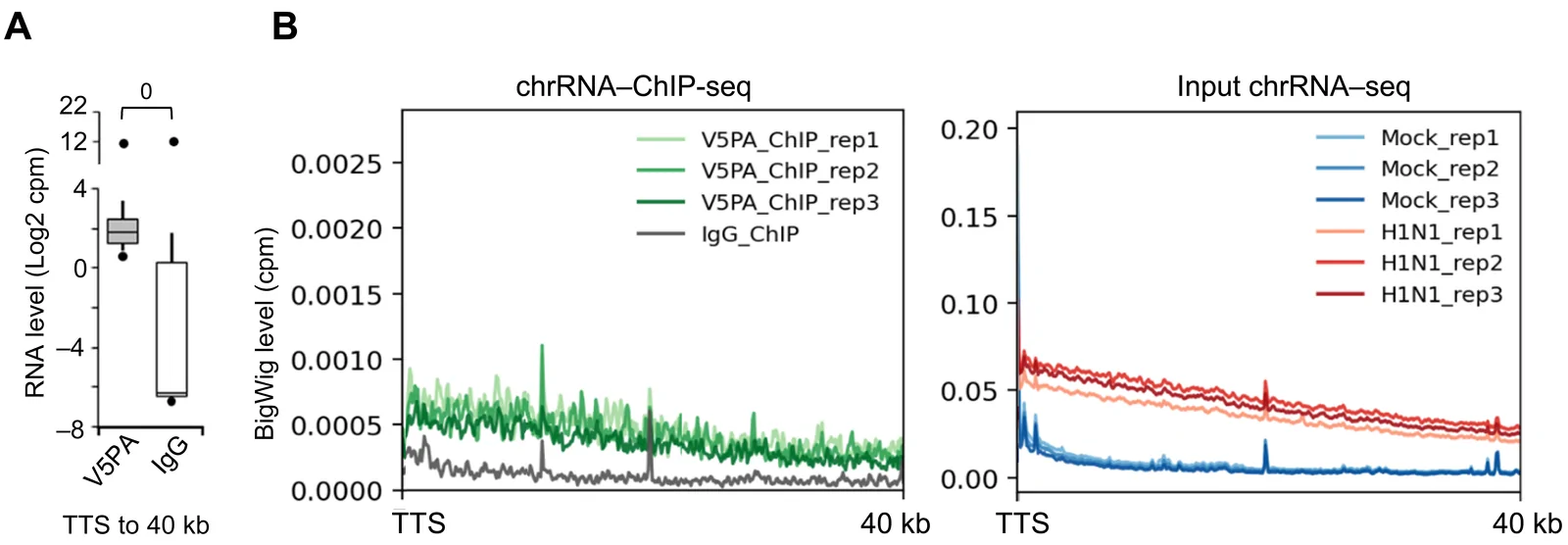
(**A**) Chromatin-associated RNA from infected A549 cells was immunoprecipitated using V5 or IgG antibodies and analyzed by deep sequencing. For each selected gene, DoG levels were quantified across a 40 kb window downstream of the transcription termination site (TTS) and expressed as log2(cpm). Only windows containing ≥ 5 reads that did not overlap with neighboring genes were included in the analysis. Data are presented as box plots, formatted similarly to Figure 2B. (**B**) Metaplot coverage profiles across the 3′ extended genomic regions. Normalized coverage profiles (cpm) were generated from BigWig files across the 40 kb region downstream of the TTS for 4900 selected genes lacking neighboring downstream genes and filtered to remove repetitive elements that cause signal noise. Left panel shows V5-PA chrRNA-ChIP-seq signal (green) compared to IgG control (gray). Right panel displays corresponding input chrRNA-seq coverage in virus-infected (red) and mock-infected (blue) cells.

## Data Availability

The data presented in this study are openly available in NCBI GEO at https://www-ncbi-nlm-nih-gov.insb.bib.cnrs.fr/geo/query/acc.cgi?acc=GSE218084 (accessed on 8 September 2026), reference number GSE218084.

## Supplementary Materials

The following supporting information can be downloaded at: https://www.mdpi.com/article/10.3390/v18101063/s1, Excel spreadsheet containing, in separate sheets, the underlying numerical data, statistical analysis and lists of genes used in pathway analysis for figures. Table S1: Chromatin-bound RNAs and differential RNA levels; RNAs bound to chromatin and differential RNA levels (related to Figure 1A–C and Figure A1A,C); Table S2: Gene list and Gene Ontology Analysis and Molecular Signature Hallmark for 6928 genes upregulated >4 (FDR < 0.05), 1957 genes downregulated >1.5 (FDR < 0.05). Note: 32,344 expressed genes (all levels) were used as background genes to calculate adjusted *p*-values of pathways using Enrichr (related to Figure A1D); Table S3: Gene lists and KEGG pathways for 4034 genes with high RNA levels (Figure A4G), 2061 genes with the highest levels of H3K27ac at the TSS (Figure A4H), 1762 genes with increased levels of H3K27ac at the TSS (Figure A4I) and 1815 genes with at least 1.5-fold enrichment of PA and PB1 at the TSS (Figure 3C) further categorized into 469 genes with increased H3K27ac (Figure 3F, left), 334 with decreased H3K27ac (Figure A4K, left), 204 with downregulated RNA levels (Figure 3F right), and 387 with upregulated RNA levels (Figure A4K, right). Note: A background set of 14,962 expressed genes (all levels) with H3K27ac% in the top 90th percentile and no neighboring TSS within 5 kb were used as background genes to calculate adjusted *p*-values of pathways using Enrichr; Table S4: (A): Gene lists and KEGG pathway analysis of 3511 expressed genes (>Q1) close to 458 enhancers with >20% FluPol enrichment (related to Figure 4D right): list of the 20 closest genes in the region −500 kb to +500 kb around the peaks and list of the 24,009 genes expressed genes (>Q1) used as background genes to calculate adjusted *p*-values of pathways using Enrichr; (B): Gene lists and KEGG pathway analysis of 3947 expressed genes (>Q1) close to 459 enhancer without FluPol enrichment (related to Figure 4D left): list of the 20 closest gene in the region −500 kb to +500 kb around the peaks. The same background gene list as in Table S4A was used to calculate adjusted *p*-values of pathways using Enrichr; Table S5: Gene lists and Gene Ontology, Molecular Signature Hallmark and KEGG pathway analysis for 4054 genes with ≥20-fold enrichment of v5-PA-bound RNAs on gene bodies (Figure 5F), subdivided into 472 genes with increased and 327 genes with decreased H3K27ac at the TSS; 1096 genes with ≥1.5-fold (80% CI) enrichment of V5-PA-bound RNAs on exons (Figure 5G); 78 genes from the above group showing additional FluPol enrichment at the TSS, as defined in Figure 3C. Note: The same background gene list as in Table S2 was used to calculate adjusted *p*-values of pathways using Enrichr; Table S6: Gene lists and pathway analysis of genes with downstream enrichment; 1454 genes with ≥1.2-fold enrichment (90% CI) of V5-PA-immunoprecipitated DoG RNAs and 3410 genes without DoG enrichment (TTS to +10 kb; related to Figure 6D, left), 1676 genes with ≥1.5-fold enrichment (80% CI) of PA and PB1 (TTS to +1 kb; related to Figure 6D, right); 118 genes representing the overlap between the two groups (related to Figure 6D, middle panel). Note: A list of 9021 expressed genes (>Q1) with DoGs and no neighboring genes within the counting window (TTS to +40 kb by 10 kb bins) was used as background genes to calculate adjusted *p*-values of pathways using Enrichr; Table S7: List and sequences of qPCR primers; Table S8: Summary of high-throughput sequencing alignment statistics. Detailed sequencing read counts (raw numbers and percentages) for chrRNA-seq (inputs) and RNA-ChIP-seq samples mapped onto the human host genome (hg19) and the influenza A virus genome (H1N1). Values are expressed as percentages of unique mappers, multi-mapping reads, and total aligned reads across replicates for each condition (uninfected inputs, infected inputs, V5-PA ChIP, and IgG control ChIP). For viral genome alignment, the first 15 nucleotides at the 5′ end of the reads were trimmed prior to mapping to exclude host-derived cap-snatched leader sequences.

## Abbreviations

The following abbreviations are used in this manuscript:

ChIP	chromatin immunoprecipitation
cpm	counts per million mapped reads
CTD	C-terminal domain
DoG	downstream-of-gene RNAs
eRNA	enhancer RNA
ER	endoplasmic reticulum
FDR	False Discovery Rate calculated by the Benjamini and Hochberg procedure applied to *p*-values
FluPol	viral RNA polymerase complex
H1N1	influenza virus A/WSN/1933
H3K4me3	histone 3 trimethyled at lysine 4
H3K27ac	histone 3 acetylated at lysine 27
hpi	hours post-infection
IAV	influenza A virus
(k)bp	(kilo) base pair
PROMPT	promoter antisense RNA
S5p	phosphorylated at the serine-5
RNAPII	RNA polymerase II
rpkm	Reads Per Kilobase of transcript per Million mapped reads
rpm	revolution per minute
seq	deep sequencing
TSS	transcription start site
TTS	transcriptional termination site
